# The DEAD-box RNA helicase eIF4A is a crucial factor for stem-cell activity and reproduction of the parasite *Schistosoma mansoni*

**DOI:** 10.3389/fcimb.2025.1731808

**Published:** 2026-01-09

**Authors:** Sophie Welsch, Oliver Puckelwaldt, Sagar Ajmera, Francesca Magari, Simone Haeberlein, Arnold Grünweller, Christoph G. Grevelding

**Affiliations:** 1 Institute of Parasitology, Biomedical Research Center Seltersberg (BFS), Justus Liebig University, Giessen, Germany; 2 Institute of Pharmaceutical Chemistry, Philipps University Marburg, Marburg, Germany

**Keywords:** eIF4A, ovary, reproduction, RNA helicase, *Schistosoma mansoni*, stem cell, testis

## Abstract

**Introduction:**

The parasite *Schistosoma mansoni* has a unique reproductive biology, because female maturation depends on constant pairing with a male. Paired females produce each up to 300 eggs per day, which are the pathogenic factors of schistosomiasis, a neglected tropical disease that affects > 240 million people worldwide. Due to the importance of egg production for life-cycle maintenance and pathology, molecular mechanisms controlling schistosome reproduction are in the focus of research. Among the candidates involved in regulating the reproductive biology of this parasite are DEAD-box RNA helicases. These enzymes are associated with various cellular processes, including ribosome biogenesis and post-transcriptional regulation. In platyhelminths, helicases are largely unexplored. One member of the DEAD-box helicase family is the eukaryotic translation initiation factor 4A (eIF4A), which unwinds stable RNA structures in the 5’-untranslated region of selected mRNAs.

**Objectives:**

We functionally characterized two eIF4A isoforms of *S. mansoni* (SmeIF4A-a and SmeIF4A-b), which are potentially involved in translation initiation like their human orthologs, to evaluate their importance for parasite vitality and reproduction.

**Methodologies/findings:**

Transcripts of both SmeIF4A isoforms were localized in female ovaries as shown by whole mount *in situ* hybridization. RNA-interference (RNAi) experiments revealed a decisive role of SmeIF4A-a in gonad maintenance and egg production. Stem-cell proliferation assays and confocal laser scanning microscopy uncovered the loss of proliferation activity in germinal and somatic stem cells after Sm*eif4a-a* RNAi. No distinct function was found for SmeIF4A-b.

**Conclusion:**

Our results suggest that SmeIF4A-a is a key factor in stem-cell proliferation and gonad maintenance, and thus also in egg production.

## 1 Introduction

The family of DEAD-box RNA helicases are named after their conserved motif D-E-A-D (Asp-Glu-Ala-Asp; Linder et al., 1989; Cordin et al., 2006). In model organisms, DEAD-box RNA helicases are involved in various cellular processes such as ribosome assembly (Nishi et al., 1988), RNA splicing (Schwer and Guthrie, 1991), RNA chaperoning and RNP remodeling (Jarmoskaite and Russell, 2014), cell differentiation and growth (Hay et al., 1990), as well as translation initiation (Rozen et al., 1990). One representative helicase of this family is eIF4A, the eukaryotic translation initiation factor 4A. It unwinds stable RNA structures in the 5’-untranslated region (Rozen et al., 1990) of selected mRNAs (Wolfe et al., 2014) during cap-dependent translation initiation and thereby facilitates translation. Three eIF4A isoforms (eIF4AI, eIF4AII, eIF4AIII) have been described (Nielsen and Trachsel, 1988; Owtrim et al., 1991), which despite their relatedness differ in several aspects. eIF4AI and eIF4AII are cytoplasmic proteins and share a sequence identity of ~ 90% (Nielsen and Trachsel, 1988), whereas eIF4AIII as a nuclear protein exhibits only ~ 60% sequence identity to eIF4AI and eIF4AII (Li et al., 1999). Functionally, eIF4AIII is involved in exon-junction complex formation (Le Hir and Andersen, 2008) in contrast to eIF4AI and eIF4AII, which are part of the translation initiation complex eIF4F (Yoder-Hill et al., 1993). Although eIF4AI and eIF4AII show high sequence identity, these isoforms are expressed at different levels depending on the tissue type (Nielsen and Trachsel, 1988), and appear to be functionally distinct (Galicia-Vázquez et al., 2012; Du et al., 2025).

While eIF4A has been intensively studied regarding biochemical and functional aspects in model organisms like mouse (Nielsen and Trachsel, 1988; Williams-Hill et al., 1997; Rogers et al., 1999, Rogers et al., 2001; Galicia-Vázquez et al., 2014), *Drosophila melanogaster* (Hernández et al., 2004), *Arabidopsis thaliana* (Bush et al., 2015), and *Saccharomyces cerevisiae* (Linder and Slonimski, 1988; Montero-Lomelí et al., 2002; Venturi et al., 2018), no data are available about its presumptive role(s) in flatworms (platyhelminths). This phylum comprises several free-living and parasitic organisms. Among these is the trematode *Schistosoma mansoni*, which infects humans and animals (Colley et al., 2014; Kouadio et al., 2020). In humans, *S. mansoni* and other schistosome species cause the neglected tropical disease schistosomiasis (Group for Neglected Tropical Diseases, 2012). Schistosomes have a unique biology as they develop into separate sexes in the mammalian host. Furthermore, pairing of male and female schistosomes initiates and maintains the differentiation of female reproductive organs (Popiel and Basch, 1984; Kunz, 2001). *S. mansoni* couples produce up to 300 eggs per day, which are the pathogenic driver of the disease (Group for Neglected Tropical Diseases, 2012; McManus et al., 2018). Stem cells play an important role for schistosome development and reproduction, and they are subdivided in germinal stem cells (GSCs) and somatic stem cells (SSCs; Collins et al., 2013; Wang et al., 2013, Wang et al., 2018). While SSCs (neoblasts) are involved in the maintenance of tissues e.g., the tegument and gastrodermis (Wendt et al., 2018), and wound healing (Collins and Collins, 2016), the proliferation of GSCs in testis, ovary, and vitellarium is a prerequisite for reproduction of this parasite (Wang et al., 2018). In this context, the DEAD-box RNA helicase *vasa* was found to be required for stem-cell proliferation and maintenance in sporocysts (Wang et al., 2013) as well as adult worms (Skinner et al., 2020). In *S. mansoni*, 33 putative DEAD-box RNA helicases have been described by *in silico* analysis (Skinner et al., 2012), including helicase orthologs of eIF4A. To date, no functional studies about eIF4A isoforms have been conducted in any platyhelminth species, thus the question remains whether there are greater functional differences between platyhelminth eIF4A isoforms to those known from model organisms (Galicia-Vázquez et al., 2012; Du et al., 2025).

Here, we characterized SmeIF4A-a and SmeIF4A-b at the molecular and functional level. The obtained data suggest that SmeIF4A-a is a crucial factor for reproduction and development of this parasite, while no clear function could be assigned to SmeIF4A-b.

## 2 Materials and methods

### 2.1 Parasite maintenance

The life cycle of a Liberian strain of *S. mansoni* (Bayer AG, Monheim, Germany) was maintained by infecting *Biomphalaria glabrata* snails as intermediate hosts and Syrian hamsters (*Mesocricetus auratus*) as final hosts in accordance with the European Convention for the protection of vertebrate animals used for Experimental and other Scientific Purposes (ETS, No. 123; revised Appendix A). The experiments have been approved by the Regional Council Giessen, Germany (V54-19c 20 15h 02 GI18/10). Single-sex (ss) and bisex (bs) worms were generated by monomiracidial and polymiracidial infection of the intermediate host, respectively (Grevelding, 1999). Adult bs and ss worms were recovered by hepatoportal perfusion of the final host 46 days (d; for bs) and 67 d (for ss) post infection, respectively. Worms were transferred to petri dishes containing M199 (3+) medium (Gibco, Germany), supplemented with 10% (v/v) newborn calf serum (NCS; Sigma-Aldrich, Germany), 1% (v/v) HEPES (Carl Roth, Germany, pH 7.4), and 1% (v/v) antibiotic-antimycotic solution (ABAM; CCPro, Germany) consisting of 10,000 U penicillin, 10 mg streptomycin and 25 µg amphotericin B per ml. The parasites were incubated at 37°C and 5% CO_2_.

### 2.2 Phylogenetic analysis

Three eIF4A isoforms of *S. mansoni* were identified by Basic Local Alignment Search Tool for proteins (BLASTp) using the three known human eIF4A isoform sequences. A multiple sequence alignment of human and *S. mansoni* eIF4A sequences was generated with Clustal Ω (Madeira et al., 2024) and visualized using ESPript 3.0 (Robert and Gouet, 2014) to analyze the parasite eIF4A sequences for the presence of conserved motifs described for DEAD-box RNA helicases (Li et al., 1999; Cordin et al., 2006). The phylogenetic tree was created for grouping three eIF4A isoforms of *S. mansoni* with their potential orthologs of other platyhelminths and selected model organisms. Appropriate sequences were obtained from NCBI protein Blast (Altschul et al., 1990), the ortholog finder of WormBase ParaSite (Howe et al., 2016, Howe et al., 2017), InterProScan (Blum et al., 2025), and the UniProt database (Consortium et al., 2025). The DEAD-box RNA helicases p68 of *Mus musculus* and *Saccharomyces cerevisiae* served as outgroup. Identified orthologs and paralogs with their respective accession numbers are listed in **Supplementary Table S1**. Multiple sequence alignments were performed using MAFFT v7.520 (Katoh and Standley, 2013) with the L-INS-i parameters for less than 1,000 sequences. The alignment was further refined with MUSCLE v5.1.0 (MUltiple Sequence Comparison by Log-Expectation; Edgar, 2004). Bayesian inference analysis was performed using MrBayes v3.2.7a (Ronquist et al., 2012) with the LG+I+G (Le and Gascuel + invariable sites + gamma distributed rate heterogeneity) model and fixed state frequencies. Twenty-five percent of the 1,000,000 generated trees were excluded as burn-in, whereas the remaining 75% were utilized to construct the majority-rule consensus tree. The generated phylogenetic tree was visualized by the interactive tool Tree Of Life (iTOL; Letunic and Bork, 2024).

### 2.3 RNA isolation and reverse transcription quantitative (real time) PCR

For RNA isolation, *S. mansoni* couples were separated by incubation with 0.26% (w/v) ethyl 3-aminobenzoate methanesulfonate (Sigma-Aldrich, Germany) dissolved in M199 (3+) medium, as described elsewhere (Collins et al., 2013). Males and females were collected separately in 1.5 ml reaction tubes, and then washed twice with PBS (pH 7.4). Supernatants were replaced by DNA/RNA protection reagent (New England Biolabs, NEB; UK) and the worms immediately frozen in liquid nitrogen and stored at -80°C until further use. After thawing on ice, tissue was mechanically homogenized and RNA isolated using the Monarch^®^ Total RNA Miniprep Kit (NEB, UK) according to the manufacturer’s instructions. Concentration and integrity of isolated RNA were determined by electropherograms (Bioanalyzer 2100; Agilent Technologies, USA) using the Agilent 6000 Nano or Pico kit. Synthesis of cDNA was performed with 200 ng and 100 ng RNA for males and females, respectively, with the QuantiTect^®^ Reverse Transcription Kit (Qiagen, Germany). Transcript levels were defined by RT-qPCR and amplified by gene-specific and exon-spanning primers. The primers were analyzed for the absence of primer dimers and only used if their efficiencies ranged between 1.8 – 2.2 (**Supplementary Table S2**). The final reaction consisted of 10 µl 2x KAPA SYBR^®^ Fast qPCR Master Mix (Kapa Biosystems, USA), 0.8 µl forward and reverse primer (10 µM, each), 3.4 µl PCR-grade water (Carl Roth, Germany), and 5 µl cDNA (1:20 diluted for male, 1:10 dilution for female samples). Cycling conditions were as follows: initial denaturation at 95°C for 3 min, followed by 45 cycles of denaturation at 95°C for 10 s, annealing at 60°C for 15 s, and elongation at 72°C for 20 s. Final elongation was performed at 60°C for 3 min. Amplification was followed by a melt curve analysis ranging from 60°C to 95°C with a stepwise increase of 1°C for 20 s to confirm amplification of a single product and the absence of primer dimers (Rotor Gene Q with Q-rex software, Qiagen, Germany). Relative transcript levels after RNAi were calculated by the Pfaffl method (Pfaffl, 2001) and are shown as log(2) fold changes. Sm*letm1* is a housekeeping gene of *S. mansoni* and was used as reference gene for all RT-qPCR analyses, as described before (Haeberlein et al., 2019). Transcript profiles of SmeIF4A-a and SmeIF4A-b were analyzed in bM (males obtained from couples after separation), sM (males obtained from ss infections, without any previous pairing experience), bF (females obtained from couples after separation), and sF (females obtained from ss infections, without any previous pairing experience), and were calculated using the Pfaffl method (Pfaffl, 2001). Sm*letm1* (Haeberlein et al., 2019) was used for normalization, and transcript profiles are shown as relative transcript levels.

### 2.4 Cloning of gene-specific fragments for riboprobe and dsRNA synthesis

Cloning of gene-specific fragments in the T7, T3, and SP6 promotors-containing pJC53.2 plasmid (Collins et al., 2010) was done as follows. By polymerase chain reaction (PCR), gene-specific amplicons of Sm*eif4a-a* and Sm*eif4a-b* of 400–500 bp (**Supplementary Table S3**) were generated. The Accu Prime *Taq* DNA Polymerase High Fidelity kit (Invitrogen, USA) was used to amplify PCR products with 3’ A overhangs. The reaction was set up according to the manufacturer’s instructions with 10 ng of plasmids carrying the coding sequences of Sm*eif4a-a* and Sm*eif4a-b*, respectively, 1 µl of corresponding primers (10 µM, each), and 1.5 µl ethylene glycol (100%; Merck, Germany) in a 50 µl reaction. The initial denaturation at 94°C for 2 min was followed by 34 cycles of denaturation at 94°C for 30 s, annealing at 58°C for 30 s, and elongation at 68°C for 1 min. A final elongation was performed at 68°C for 5 min (C1000™ Thermal Cycler, Bio-Rad, USA). PCR products were analyzed by agarose gel electrophoresis and extracted from the gel using the Monarch^®^ DNA Gel Extraction Kit (NEB, UK). Amplicons were recovered in 15 µl elution buffer. The pJC53.2 plasmid was linearized by the restriction enzyme *Ahd*I (NEB, UK), which resulted in T overhangs. Inserts were ligated into pJC53.2 in 3:1 ratio using T4 ligase (NEB, UK) at 4°C overnight. Transformation of chemically competent *Escherichia coli* of the NEB 5α strain (NEB, UK) was performed by incubating 10 µl of the ligation reaction with 100 µl *E. coli* cells for 30 min on ice. A heat shock was applied for 80 s at 42°C followed by 5 min on ice before 900 µl of lysogeny broth medium (LB; Carl Roth, Germany) were added. Cells were incubated for 1 h at 37°C shaking and centrifuged for 1 min at 900 g. Approximately 900 µl of the supernatant were discarded, and cells were resuspended in the remaining volume of 100 µl. Transformed *E. coli* were selected on kanamycin (50 µg/ml; Sigma-Aldrich, Germany) containing LB agar plates and incubated at 37°C overnight. The sequence of integrated inserts into pJC53.2 was verified by Sanger sequencing (Microsynth SeqLab, Germany). The created constructs were used for riboprobe and dsRNA synthesis.

### 2.5 Riboprobe and dsRNA synthesis

Gene-specific PCR amplicons of approximately 500 bp (**Supplementary Table S3**), which derived from the pJC53.2 plasmid constructs, were used to synthesize dsRNA for RNA interference (RNAi) experiments or for synthesizing riboprobes for whole mount *in situ* hybridization (WISH). Additionally, a non-schistosomal dsRNA encoding the *E. coli* ampicillin resistance gene (*ampR*) was synthesized as a control for RNAi as described elsewhere (Moescheid et al., 2023). Inserts of the pJC53.2 construct were amplified with a primer specific for the T7 promotor sequence (*T7_extended* 5’-*CCT AAT ACG ACT CAC TAT AGG GAG*-3’) in a 50 µl reaction consisting of: 8 ng plasmid, 2 µl dNTPs (10 mM each; Solis BioDyne, Estonia), 6 µl T7_extended primer (10 µM), 1 µl Betaine (5 M; Sigma-Aldrich, Germany), 10 µl Q5 reaction buffer (NEB, UK), and 0.5 µl Q5 High-Fidelity Polymerase (NEB, UK). Samples were first denatured at 98°C for 2 min, followed by 35 cycles of denaturation at 95°C for 15 s, annealing at 64°C for 20 s, and elongation at 72°C for 40 s. The final elongation occurred at 72°C for 3 min. PCR success was confirmed by agarose gel electrophoresis. For riboprobe synthesis, amplicons were purified with the Monarch^®^ Spin PCR & DNA Cleanup Kit (NEB, UK). *In vitro* transcription was modified after Collins et al., 2010. In brief, approximately 0.5 µg PCR product was used in a reaction with 20 µl rNTP mix (25 mM each; NEB, UK), 10 µl reaction buffer (10x), 1 µl inorganic pyrophosphatase (NEB, UK), 10 µg T7 RNA polymerase (expression vector kindly provided by Prof. Collins, UT Southwestern Medical Center), and Diethylpyrocarbonate (DEPC)-treated water to a final volume of 100 µl. The reaction was incubated overnight at 37°C. Remaining template DNA was removed by incubation with 10 U DNase I (NEB, UK) at 37°C for 30 min. Synthesized dsRNA was precipitated with 100 µl lithium chloride (7.5 M; Merck, Germany) at -20°C for at least 1 h. Samples were centrifuged for 30 min at 4°C, top speed. The supernatant was removed, and the pellet washed with 500 µl ice-cold ethanol (70%). Samples were centrifuged for 3 min at 4°C, top speed. Ethanol was cleaned from the reaction, and the dsRNA pellet resuspended in DEPC-treated water. For riboprobe synthesis, *in vitro* transcription of 0.5 µg PCR product comprising the T3 and SP6 promotors was performed to synthesize single-stranded RNA (ssRNA) probes. To this end, a Digoxigenin-11-UTP (DIG-UTP) rNTP mix was prepared by mixing 5 µl ATP, CTP, GTP (100 mM each; NEB, UK) with 3.5 µl UTP (100 mM; NEB, UK), and 17.5 µl DIG-UTP (10 mM; Roche, Switzerland) and filled up to 50 µl with DEPC-treated water. The *in vitro* transcription reaction consisted of: 0.5 µg PCR product, 1 µl T3 or SP6 RNA polymerase (Roche, Switzerland), 2 µl transcription buffer (10x; Roche, Switzerland), 2 µl DIG-rNTP mix, 0.6 µl murine RNase inhibitor (NEB, UK) in a final volume of 20 µl. Samples were incubated at 28°C for approximately 16 h. Template DNA was digested by 10 U RNase-free DNase I (NEB, UK) for 20 min at 37°C. An equal volume of 7.5 M lithium chloride was added, and the mixture incubated at -80°C for at least 30 min. Samples were centrifuged at 16,000 g for 30 min at 4°C. The supernatant was replaced by ice-cold ethanol (70%), and the sample centrifuged again at 16,000 g for 3 min at 4°C. Ethanol was removed and the pellet resuspended in 20 µl DEPC-treated water. The concentrations of dsRNA and ssRNA-probes were photometrically determined and their integrity confirmed by agarose gel electrophoresis.

### 2.6 Transcript localization

WISH was performed for transcript localization of Sm*eif4a-a* and Sm*eif4a-b*, using a previously established protocol (Cogswell et al., 2011; Collins and Collins, 2017; Li et al., 2024; Moescheid et al., 2025) with the following modifications. Bs male tissue was permeabilized with 45 µg/ml proteinase K (Ambion, UK) in 1x PBSTx (PBS in DEPC-treated water + 0.3% TritonX-100; Sigma-Aldrich, Germany), and tissue of bs females with 20 µg/ml for 45 min at room temperature (RT) with moderate agitation. For hybridization, the worms were incubated with 200 ng/ml (Sm*eif4a-a*, Sm*vlg1*, Sm*myst4*) or 20 ng/ml (Sm*eif4a-b*, Sm*tsp-2*) of the appropriate riboprobe in a final volume of 300 µl at 55 °C and 135 rpm (GFL, Germany) for at least 16 h. The probe concentration of Sm*eif4a-b* and Sm*tsp-2* was adjusted to reduce background signals. Worm samples were incubated with an anti-DIG-AP antibody (11093274910; Roche, Switzerland) diluted 1:2,000 in colorimetric block solution [7.5% heat-inactivated horse serum (H1138; Sigma-Aldrich, Germany) in Tris-NaCl-Tween-20 buffer solution] at 4°C overnight to detect target-bound riboprobes. Signals developed after the addition of the substrates nitro blue tetrazolium (11383213001; Roche, Switzerland) and 5-bromo-4-chloro-3-indolylphosphate (11383221001; Roche, Switzerland). Development was directly performed at RT for Sm*eif4a-a*, Sm*vlg1*, and Sm*myst4*, while Sm*eif4a-b* and Sm*tsp-2* samples were incubated in AP buffer with added substrates at 4°C overnight and signals continued to be developed the next day at RT. Signal development was stopped at different time points by transferring worms in PBSTx. Worms were finally embedded on slides in 80% glycerol and imaged using an inverted laboratory microscope (DM IL LED; Leica Microsytems, Germany) with the WaveImage software (VWR, USA).

### 2.7 RNAi

Transcriptional knockdowns (KD) of Sm*eif4a-a* and Sm*eif4a-b* were performed by RNAi. To this end, gene-specific dsRNAs were synthesized and the worms incubated in a modified version of the ABC169 medium (Wang et al., 2019) for soaking, as described before (Li et al., 2024; Moescheid et al., 2025). In brief, Basch medium (Basch, 1981) was supplemented with 10% (v/v) NCS, 1% (v/v) ABAM, 0.2% (v/v) red-blood cells (10% suspension; Biochrome, Germany), 200 µM ascorbic acid (Sigma-Aldrich, Germany), and 0.25% (v/v) low-density lipoprotein (LDL; Trina Bioreactives, Switzerland). Worms were incubated in 6-well plates (Greiner Bio-One, Germany) with 5 ml prewarmed ABC169 medium and 15 *S. mansoni* couples per well and kept in an incubator (nunc™ Galaxy S+; RS Biotech, Germany) at 37°C and 5% CO_2_. Each approach was performed as technical duplicate, and worms deriving from different hamsters served as biological replicates. In total, six biological replicates were analyzed. DsRNAs against the target genes or the control were added one day after perfusion at a concentration of 15 µg/ml (Sm*eif4a-a*) or 10 µg/ml (Sm*eif4a-b*), respectively. Pilot experiments were performed with varying concentrations of dsRNA for each gene to determine the optimal concentration to reach sufficient knockdown efficiencies. However, these experiments were conducted in a different medium (M199 3+), and knockdown efficiencies might vary depending on the medium composition (Štefanic et al., 2010). Controls consisted of irrelevant dsRNA (*ampR*) of the same concentration and a control without dsRNA but adding an equal volume of DEPC-treated water to the parasite culture. Worms were transferred into new 6-well plates filled with prewarmed ABC169 medium 24 h after the start of the experiment using feather-weight tweezers alongside with the addition of dsRNA or DEPC-treated water. This procedure was repeated every 2–3 d until d 21. Phenotypes were investigated by an inverted laboratory microscope (DM IL LED; Leica Microsytems, Germany) considering the pairing status (paired or separated), worm attachment to the petri dish, worm motility, egg production, and morphological changes. Worm motility was classified as 0 = total absence of movement; 1 = minimal movement restricted to the gut, head or tail region; 2 = reduced motility; 3 = normal motility; 4 = hyperactivity according to Ramirez et al., 2007; Keiser, 2010; Manneck et al., 2010. *In vitro* laid eggs were counted and grouped as normal or abnormal eggs with respect to their morphology. Abnormal eggs were classified based on alterations in shape (e.g. absence of the spine) or a reduced egg size.

### 2.8 Lipid staining

The vitellarium of *S. mansoni* bs females consists of fully differentiated, lipid-rich vitellocytes (Kunz, 2001; Lu et al., 2015). In order to investigate RNAi effects on mature vitellocytes, lipid droplets were stained by a modified version of the Oil-Red O staining method (Anthony et al., 2010; Wang et al., 2019). After 21 d of RNAi, couples were separated by 0.26% (w/v) ethyl 3-aminobenzoate methanesulfonate (Sigma-Aldrich, Germany) dissolved in M199 (3+), as described before (Collins et al., 2013). Then, worms were fixed and stored in 2% paraformaldehyde (Carl Roth, Germany) in 1x PBSTx at 4°C until further use. Next, the worms were washed twice in 1x PBSTx and transferred into netwell inserts (Science Service) fitting into individual wells of a 12-well plate. Worms were incubated in 99% propane-1,2-diol (Merck, Germany) for 5 min at RT, while shaking at 135 rpm (10 worms/ml). The solution was replaced by 0.5% (w/v) Oil-Red O (Sigma-Aldrich, Germany) dissolved in propane-1,2-diol, and staining occurred for 45 min at RT on a shaker. Afterwards, worms were washed twice with 85% propane-1,2-diol for 5 min each at RT with agitation. A final washing step was performed with 1x PBS until worms were embedded with ROTI Mount FluorCare (Carl Roth, Germany). The worms were immediately analyzed by phase contrast microscopy using an inverted laboratory microscope (DM IL LED; Leica Microsytems, Germany). The size of the vitellarium (mm^2^) was determined with ImageJ using the selection brush tool (Schindelin et al., 2012; Schneider et al., 2012; Rueden et al., 2017).

### 2.9 EdU cell-proliferation assay and confocal laser scanning microscopy

To investigate stem-cell proliferation after 21 d of RNAi, 5-ethynyl-2’-deoxyuridine (EdU) was added to the worms *in vitro* at a final concentration of 10 µM (Thermo Fisher Scientific, USA) for 24 h at 37°C and 5% CO_2_. Treated and control couples were separated by 0.26% (w/v) ethyl 3-aminobenzoate methanesulfonate (Sigma-Aldrich, Germany) dissolved in M199 (3+) as described before (Collins et al., 2013). Subsequently, worms were collected in 2 ml reaction tubes and fixed in 4% paraformaldehyde in 1x PBSTx at 4°C overnight. For storage, worms were rinsed in PBSTx for 3 min shaking at RT, followed by dehydration in 50% methanol (MeOH; Carl Roth, Germany) in PBSTx and 100% MeOH for 10 min each on a shaker. Samples were stored in fresh 100% MeOH at -20°C. The worms were rehydrated in 50% MeOH in PBSTx at RT on a rocker for 10 min and subsequently washed in PBSTx for 10 min. Thereafter, worms were transferred to small sized baskets (Intavis Bioanalytical instruments, Germany) fitting into single wells of a 48-well plate. Bleaching occurred for 30 min under bright light in 1.2% (v/v) H_2_O_2_ (Carl Roth, Germany), 5% (v/v) formamide (Carl Roth, Germany), 0.5x saline-sodium citrate (SSC) pH 7.0 in DEPC-treated water. After bleaching, the worms were permeabilized with 6 µg/ml proteinase K (Ambion, UK) in PBSTx for 45 min. Post fixation was carried out with 4% paraformaldehyde in 1x PBSTx for 10 min at RT. Two washing steps followed with 1x PBS for 5 min each. Staining was performed using the Click-iT Plus EdU Alexa Fluor 488 imaging kit (Thermo Fisher Scientific, USA) for 30 min with shaking. From this point on, samples were protected from light. The worms were washed twice with PBS for 5 min each at RT on a shaker before counterstaining the parasites overnight with 10 µM Hoechst 33342 (Sigma-Aldrich, Germany) in PBSTx at 4°C with shaking. Next, the worms were washed three times with PBSTx at RT for 10 min each with shaking followed by mounting on slides, as described previously (Hahnel et al., 2014). In order to quantify cell proliferation in the immature part of the ovary or the testicular lobes, where gonadal stem cells mostly occur in schistosomes (Collins and Collins, 2017; Li et al., 2021), the surface areas were determined using the selection brush tool of ImageJ (Schindelin et al., 2012; Schneider et al., 2012; Rueden et al., 2017). Proliferating EdU-positive (EdU^+^) oogonia and spermatogonia were counted, and the ratio of proliferating cells per mm^2^ was calculated, as described elsewhere (Moescheid et al., 2025).

After 21 d of RNAi, morphological changes in the reproductive organs of males and females were investigated by confocal laser scanning microscopy (CLSM) as described before (Machado-Silva et al., 1994; Neves et al., 2005; Beckmann et al., 2010). For this, couples were separated by 0.26% (w/v) ethyl 3-aminobenzoate methanesulfonate (Sigma-Aldrich, Germany) dissolved in M199 (3+). Males and females were fixed in alcohol formalin glacial acetic fixative (AFA) consisting of 2% acetic acid (Carl Roth, Germany), 1.1% paraformaldehyde, and 66.7% ethanol for 24 h at RT. The parasites were transferred to netwell inserts fitting into single wells of a 12-well plate, and staining was performed on a shaker at RT with Certistain carmine red (Merck, Germany) for 1 h. The worms were destained up to five times in acidic ethanol [70% (v/v) ethanol, 2.5% (v/v) hydrochloric acid (Carl Roth, Germany)] for 5 min each. Thereafter, worms were dehydrated by increasing ethanol concentrations (80%, 90%, 100%) for 5 min each, and fixed on slides in Euparal (Carl Roth, Germany). The software package “IMARIS for cell biologists” (v 8.4.2; Bitplane, Switzerland) was used to determine the volume of ovaries and testes, as described before (Tonk et al., 2020). Z-stacks of these reproductive organs, generated by CLSM, served as input data. For microscopic evaluation, the confocal microscope TCS SP5 VIS with the LAS AF software (Leica Microsystems, Germany) was used. Alexa 488 and carmine red were excited with an argon-ion laser at 488 nm, whereas Hoechst 33342 was excited at 405 nm. Background signals and optical section thickness were defined by setting the pinhole size to airy unit 1 (Kellershohn et al., 2018; Mughal et al., 2021). Z-stacks of ovaries and testes were acquired with a step size of 0.5 µm.

### 2.10 Statistics

Statistical analyses were performed using the GraphPad Prism V.8 software (GraphPad Software; San Diego, USA) applying the paired or unpaired two-tailed *t-*test for normally distributed data, determined by the Shapiro-Wilk test, or the two-tailed Mann-Whitney test for non-normally distributed data, as previously described (Moescheid et al., 2023, Moescheid et al., 2025). Volumes of ovaries and testicular lobes were statistically evaluated by the unpaired Welch’s *t*-test. Statistical differences with *p* < 0.05 were considered significant.

## 3 Results

### 3.1 Identification of eIF4A isoforms in *S. mansoni*

**Figure 1:**
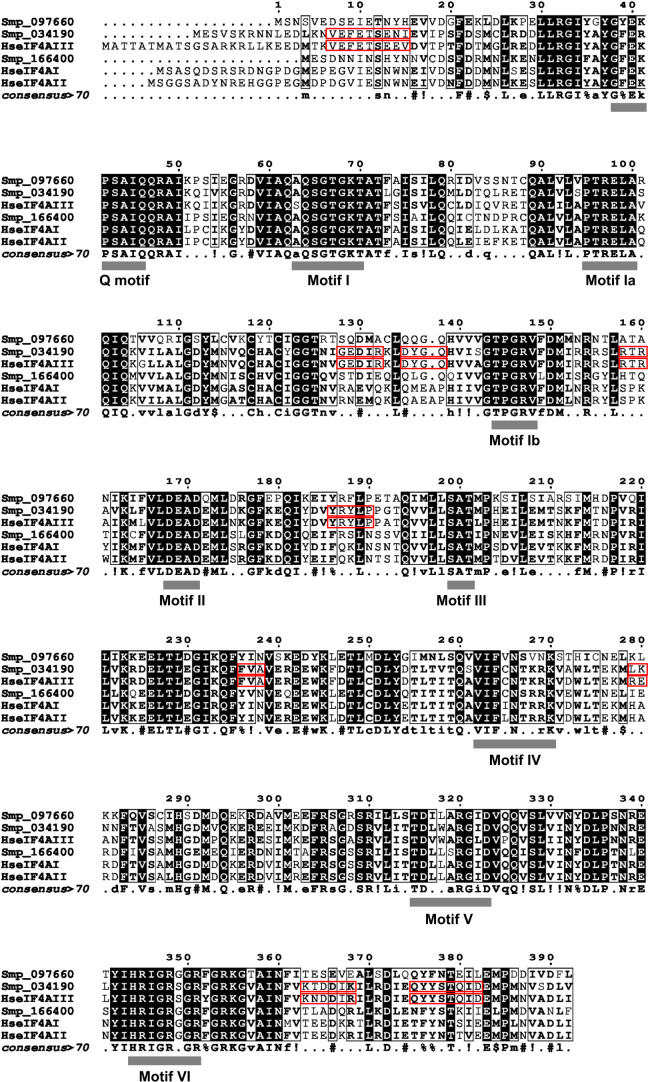
Three eIF4A isoforms were identified in *S. mansoni* by *in silico* analyses. A multiple sequence alignment of human (Hs) and *S. mansoni* (Sm) eIF4A isoforms was created with Clustal Ω (Madeira et al., 2024) and visualized using ESPript 3.0 (Robert and Gouet, 2014). Conserved motifs of eIF4A are marked with grey bars. Additional motifs of the eIF4AIII isoform are highlighted by red boxes (Li et al., 1999; Cordin et al., 2006). Uppercase letters indicate identity, lowercase letters represent consensus > 0.5;! stands for I or V, $ stands for L or M, % stands for F or Y, and # stands for N, D, Q, or E.

Three isoforms of eIF4A have been identified in *S. mansoni* by BLASTp using human orthologs as templates (accession numbers, see **Supplementary Table S1**). These SmeIF4A isoforms were analyzed for the presence of conserved motifs (Li et al., 1999; Cordin et al., 2006), as shown by multiple sequence alignment with human eIF4A sequences (**Figure 1**). All three isoforms have the Q motif and motifs I-VI, which are important for the function of the protein. The Q motif and motifs I, II, and VI are involved in ATP binding and hydrolysis, while motifs Ia, Ib, and IV are required for RNA binding (Andreou and Klostermeier, 2013). eIF4AIII can be distinguished from eIF4AI and eIF4AII due to additional conserved motifs specific for this isoform (Li et al., 1999). These motifs were found for Smp_034190 (**Figure 1**, red boxes) having a sequence identity greater than 80% to HseIF4AIII (**Figure 1**, **Supplementary Table S4**). Therefore, it was classified as *S. mansoni* eIF4AIII (SmeIF4AIII). HseIF4AIII has an amino acid (aa) sequence identity of 67% to HseIF4AI and HseIF4AII. A similar range was observed for SmeIF4A with Smp_097660 and Smp_166400 being 53% or 61% identical to SmeIF4AIII, respectively. In contrast to the high sequence identity of ~ 90% between HseIF4AI and HseIF4AII, the sequence of Smp_097660 is only to 59% identical to Smp_166400. Each of these two *S. mansoni* eIF4A isoforms exhibits a similar sequence identity to both HseIF4AI and HseIF4AII, with Smp_097660 being 57% and 58% identical to HseIF4AI and HseIF4AII, respectively, while the sequence identity of Smp_166400 to HseIF4AI and HseIF4AII was 70% in both cases (**Supplementary Table S4**). Consequently, the two SmeIF4A isoforms could not be clearly assigned as orthologs to either HseIF4AI or HseIF4AII. Since the SmeIF4A isoforms appear to be no one-to-one orthologs to the human eIF4A isoforms, they have been designated SmeIF4A-a (Smp_097660) and SmeIF4A-b (Smp_166400).

### 3.2 Phylogenetic analysis of eIF4A isoforms in platyhelminths

**Figure 2:**
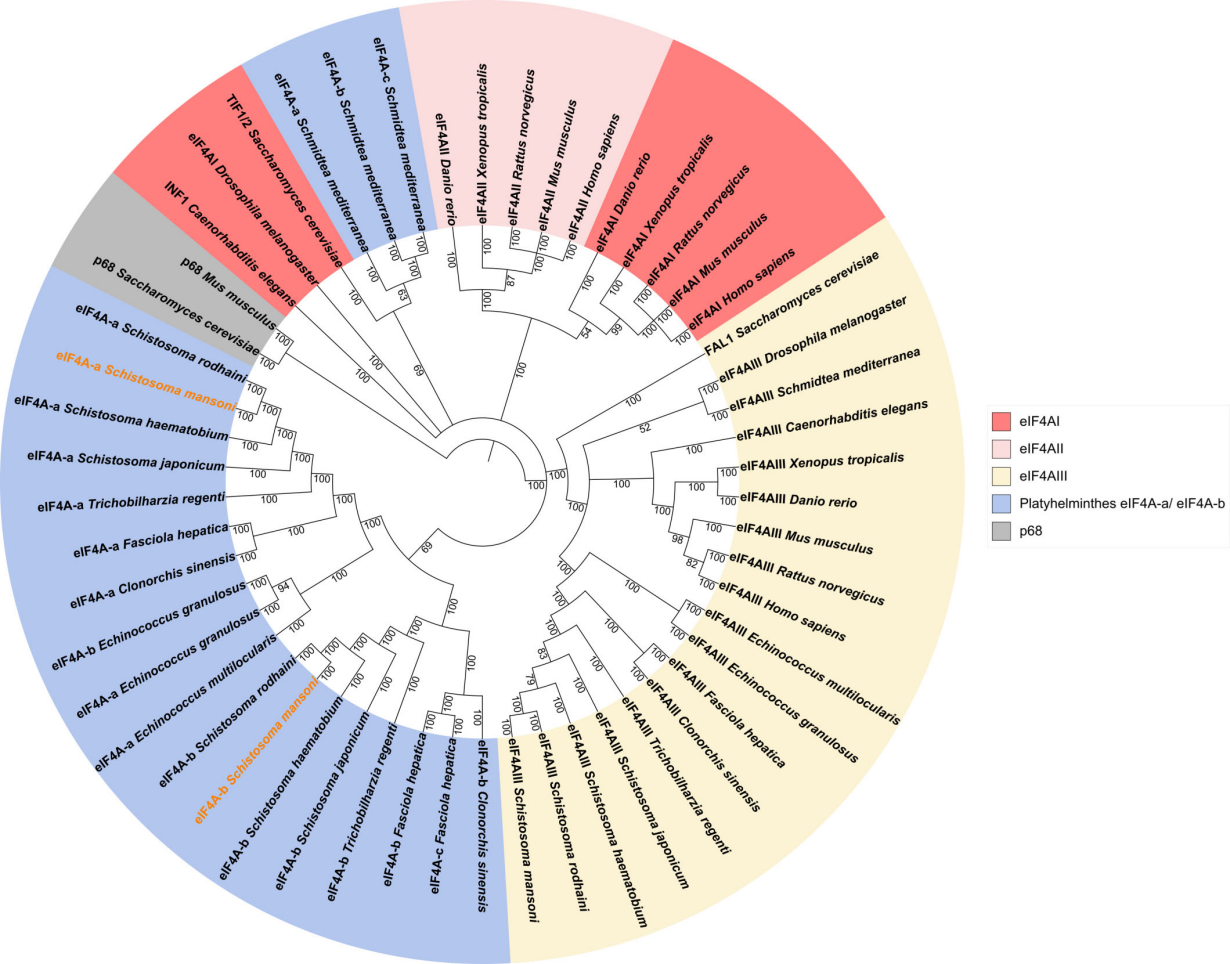
Phylogenetic analyses indicated a gene duplication of platyhelminth eIF4A that is independent from that of model organisms. Amino acid sequences of the three SmeIF4A isoforms were compared to paralogs and orthologs from model organisms and other platyhelminths. The two SmeIF4A isoforms analyzed in this study are highlighted in orange. The related DEAD-box RNA helicases p68 of *Mus musculus* and *Saccharomyces cerevisiae* served as outgroup.

Based on their aa sequences, we conducted a phylogenetic analysis for the SmeIF4A isoforms, which were compared to orthologs of organisms with different evolutionary distance, including model organisms and other platyhelminths (**Figure 2**). The DEAD-box RNA helicase p68, which is closely related to Vasa proteins, served as outgroup (Skinner et al., 2012). The resulting phylogenetic tree showed clustering of eIF4AI and eIF4AII isoforms of model organisms, consistent with the fact that they share approximately 90% sequence identity (Nielsen and Trachsel, 1988). In platyhelminths, two (or more) eIF4A genes (eIF4A-a, eIF4A-b) were identified that were not classified as eIF4AIII, and they clustered in a subgroup. The eIF4AIII isoform contains additional conserved aa motifs, which distinguish it from eIF4AI and eIF4AII isoforms (Li et al., 1999). eIF4AIII of model organisms was found to be distant to eIF4AI and eIF4AII, and it grouped together with eIF4AIII of platyhelminths. As expected, eIF4A-a, eIF4A-b, and eIF4AIII sequences from trematodes were evolutionary closely related, but distinct from the cestode *Echinococcus*. Our results confirm previous phylogenetic analyses that identified *S. rodhaini* as the closest relative of *S. mansoni* and *S. japonicum*, a member of the Asian clade, as the most distant relative (Lawton et al., 2011). The corresponding branch lengths of this phylogenetic tree are illustrated in **Supplementary Figure S1**. Additionally, we performed individual multiple sequence alignments for all eIF4A isoforms of each species (**Table 1**). The results showed a sequence identity of approximately 90% between eIF4AI and eIF4AII of vertebrates, while eIF4AIII is different with about 60% sequence identity to eIF4AI (Nielsen and Trachsel, 1988; Li et al., 1999). In platyhelminths, the sequence identity between the eIF4A-a and eIF4A-b isoforms was lower (~ 60%), and it is only slightly higher than the similarity between eIF4AIII and eIF4A-a of platyhelminths (~ 55%; **Table 1**). This indicates that the platyhelminth eIF4A-a and eIF4A-b are no one-to-one orthologs of the vertebrate isoforms eIF4AI and eIF4AII. Thus, the gene duplication of eIF4A in platyhelminths appears to be independent from that of vertebrates.

**Table 1:** eIF4A-b isoforms show lower sequence identity to eIF4A-a isoforms in platyhelminths compared to the isoform eIF4AI and eIF4AII in model organisms. Sequence analyses showed that the platyhelminth paralogs eIF4A-a and eIF4A-b are more divergent to each other than the paralogs eIF4AI and eIF4AII of model organisms. Sequence identities between eIF4AI/eIF4A-a and eIF4AII/eIF4A-b or eIF4AI/eIF4A-a and eIF4AIII of each species were analyzed by pairwise alignments using Clutal Ω.

eIF4AI/eIF4A-a	eIF4AII/eIF4A-b	eIF4AIII
*H. sapiens*	89.66%	66.91%
*M. musculus*	89.66%	66.67%
*R. norvegicus*	89.66%	66.91%
*X. tropicalis*	90.39%	67.49%
*D. rerio*	88.67%	67.25%
*D. melanogaster*	na	67.42%
*C. elegans*	na	61.90%
*S. cerevisiae*	100%	55.08%
*S. mediterranea*	na	na
*E. multilocularis*	na	56.28%
*E. granulosus*	na	56.99%
*F. hepatica*	61.17%/56.49%	57.69%
*C. sinensis*	61.66%	57.58%
*T. regenti*	58.66%	54.59%
*S. haematobium*	58.16%	53.96%
*S. japonicum*	60.00%	54.08%
*S. rodhaini*	58.45%	52.86%
*S. mansoni*	58.61%	53.32%

na, not applicable.

### 3.3 Expression and localization of two eIF4A isoforms in *S. mansoni*

**Figure 3:**
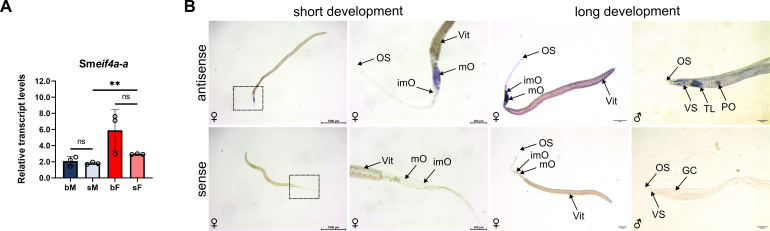
Sm*eif4a-a* transcripts occur in adult *S. mansoni* and preferentially in the female ovary. **(A)** Transcript levels were determined by RT-qPCR in pairing-experienced (bM, bF) and pairing-unexperienced (sM, sF) males and females, respectively. The housekeeping gene Sm*letm1* was used for normalization (Haeberlein et al., 2019). Significantly higher transcript levels were found in sF compared to sM. **p < 0.01 determined by *t*-test. Means and standard deviation (SD) of three independent experiments (n = 3) are shown. **(B)** Transcripts of Sm*eif4a-a* were preferentially localized in the posterior ovary of bF by WISH after short development time (upper row, left). A long signal development revealed additional signals in the anterior ovary and in the vitellarium of females, and in the testicular lobes and in muscles and neurons of males (upper row, right). The gene-specific sense probe, after short and long development, respectively, served as control and demonstrated the absence of any signal (lower row). Pictures on the left show overviews, while close-ups of the framed areas are also presented. GC = gynaecophoric canal, imO = immature part of the ovary, mO = mature part of the ovary, OS = oral sucker, PO = pseudo-ovary, TL = testicular lobes, Vit = vitellarium, VS = ventral sucker. Representative images of 3 independent experiments (n = 3) with 5–6 worms per experiment are shown.

Previous bulk RNA-seq data showed transcript levels of genes of interest in males and females before and after pairing and in their corresponding isolated gonads (testis, ovary; Lu et al., 2016, Lu et al., 2018). This data set indicated a higher transcript level of Sm*eif4a-a* in females compared to males, and slightly more transcripts in bF after pairing compared to sF as well as higher transcript levels in ovaries of bF compared to ovaries of sF (**Supplementary Figure S2**, Lu et al., 2016, Lu et al., 2018). RT-qPCRs with cDNA obtained from bM, bF as well as sM, sF, respectively, supported previous RNA-seq data with a similar tendency of slightly, but not significantly higher expression levels in females after pairing. In contrast to previous RNA-seq data, we observed significantly higher transcript levels in sF compared to sM using RT-qPCR (**Figure 3A**). Next, we performed WISH to localize Sm*eif4a-a* transcripts using a gene-specific single-stranded riboprobe. The sense probe served as negative control. As positive controls, we used previously published riboprobes of Sm*vlg1* (Skinner et al., 2012), Sm*tsp-2* (Cogswell et al., 2011), and Sm*myst4* (Li et al., 2024; **Supplementary Figure S3**). Already after short signal development (~ 30 min), Sm*eif4a-a* transcripts were detected in the middle and posterior parts of the bF ovary, where intermediate and mature oocytes are located (**Figure 3B**). After longer signal development (> 2 h), additional signals were found in the anterior part of the bF ovary, the location of oogonia, and in the vitellarium of females. Moreover, at this later time point we found signals of Sm*eif4a-a* transcripts in the testis and in striping patterns along the male body, which could be indicative for muscle and neuronal cells (Wendt et al., 2020; Li et al., 2024). These results confirmed single-cell (sc) RNA-seq data from adults, which revealed high transcript levels in the germline of bF and bM (**Supplementary Figure S4**, Wendt et al., 2020). In a recent scRNA-seq study of oocytes, Sm*eif4a-a* transcripts were found to be highly expressed in germ cells/germ cell progeny, intermediate, and late germ cells in ovaries of bF, but to a lesser extent in the somatic cell cluster (**Supplementary Figure S5**, Moescheid et al., 2025).

**Figure 4:**
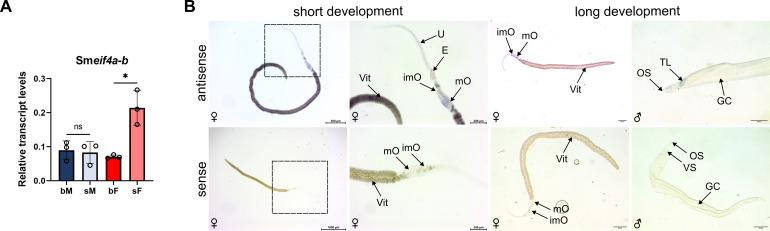
Transcript levels of Sm*eif4a-b* decreased after pairing in females and were localized in various tissues. **(A)** RT-qPCR analysis revealed significantly higher transcript levels in pairing-unexperienced (sF) compared to pairing-experienced females (bF). No difference was found between the corresponding male samples (sM, bM). Relative transcript levels were normalized using the housekeeping gene Sm*letm1* (Haeberlein et al., 2019). *p < 0.05, determined by *t*-test. Means ± SD of three independent experiments (n = 3) are shown. **(B)** WISH localized transcripts mainly in the ovary but also along the uterus and in the vitellarium of females after short and long development times (upper row). After long signal development, transcripts were localized in the testicular lobes of males (upper row, right). The gene-specific sense probe, after short and long development, respectively, served as control and demonstrated the absence of any signal (lower row). Pictures on the left show overviews, while close-ups of the framed areas are presented next to them. E = egg, GC = gynaecophoric canal, imO = immature part of the ovary, mO = mature part of the ovary, OS = oral sucker, TL = testicular lobes, U = uterus, Vit = vitellarium, VS = ventral sucker. Representative images of 3 independent experiments (n = 3) with 5–6 worms per experiment are shown.

Bulk RNA-seq data indicated that Sm*eif4a-b* may not be gender- or pairing-dependently expressed in adults (**Supplementary Figure S2**; Lu et al., 2016; Lu et al., 2018). In contrast, our RT-qPCR data revealed significantly higher transcript levels in sF compared to bF, whereas no difference was detected between males with and without pairing experience (**Figure 4A**). WISH localized transcripts of Sm*eif4a-b* in both parts of the ovary, along the uterus, and in the vitellarium of bF after short and long signal developments. Longer signal developments localized Sm*eif4a-b* transcripts in the testis and in muscles and neurons, but weaker compared to Sm*eif4a-a* (**Figure 4B**). ScRNA-seq analysis identified Sm*eif4a-b* transcripts in the GSC progeny, neurons, and muscles. However, there was no significant enrichment of Sm*eif4a-b* in any cluster (**Supplementary Figure S4**, Wendt et al., 2020). ScRNA-seq of oocytes indicated that Sm*eif4a-b* transcripts are present in the GSC/GSC progeny and intermediate stage oocytes (**Supplementary Figure S5**, Moescheid et al., 2025). Signals detected along the uterus might represent the muscles surrounding the uterus (Mair et al., 2000).

### 3.4 Functional characterization of Sm*eif4a-a* indicates a major role in gonad maintenance

**Figure 5:**
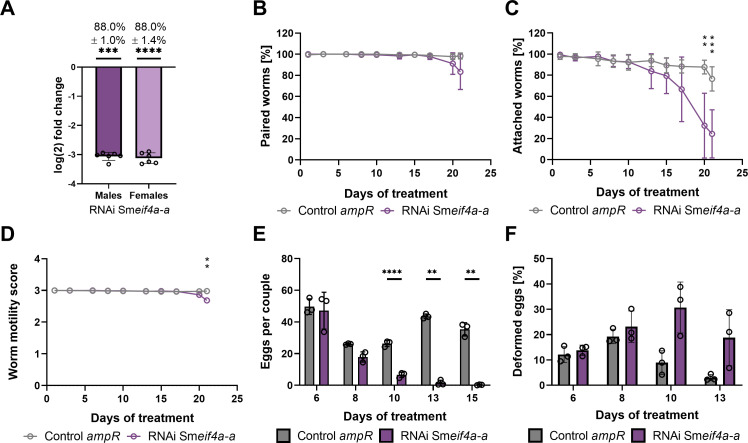
Sm*eif4a-a* RNAi decreased worm vitality and egg production. *S. mansoni* couples were treated with 15 µg/ml Sm*eif4a-a* dsRNA (purple) every 2 to 3 d, or they were incubated with *ampR* dsRNA (15 µg/ml) as irrelevant dsRNA control (grey) over a period of 21 d **(A)** Knockdown efficiency was determined by RT-qPCR measuring relative transcript levels in males and females after separation of the couples. The housekeeping gene Sm*letm1* was used for normalization (Haeberlein et al., 2019). The following phenotypes were found in the Sm*eif4a-a* RNAi group **(B)** pairing stability was constant, **(C)** worm attachment significantly decreased after 20 d, **(D)** worm motility, scored from 0 (no motility) to 4 (hyperactivity), was slightly reduced on d 21, **(E)** total egg production significantly decreased from 10 d on. Although the ratio of abnormal eggs **(F)** was not significantly different to that of the control, there was a tendency of more abnormal eggs from d 10 on. **p < 0.01, ***p < 0.001, ****p < 0.0001, determined by *t*-test. **(A-D)**, n = 6; **(E, F)**, n = 3.

**Figure 6:**
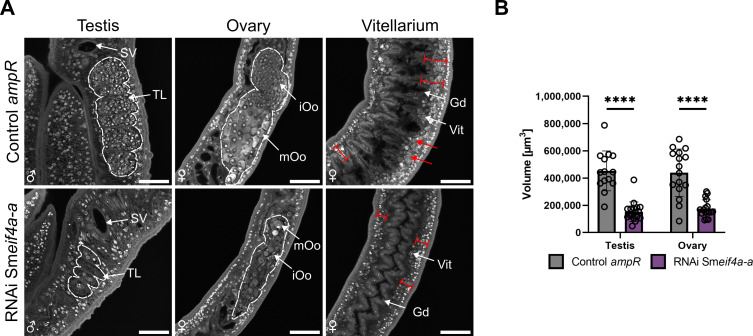
Sm*eif4a-a* RNAi impaired the morphology of reproductive organs. **(A)** CLSM analysis of carmine-red stained males and females showed reduced dimensions and less cells in the testis and ovary after Sm*eif4a-a* RNAi compared to the control (*ampR* dsRNA). The lateral dimension of the vitellarium also appeared to be diminished (red boundary line), and the number of vitellocytes was reduced upon Sm*eif4a1* RNAi. While mature vitellocytes of the stages 3/4 were observed in control worms (red arrows), they were absent in females of the Sm*eif4a-a* RNAi group. **(B)** Compared to worm gonads of the control group, the volumes of testis and ovary were significantly smaller in the Sm*eif4a-a* RNAi group. ****p < 0.0001, determined by Welch’s *t*-test. Scale bars: 50 µm. Gd = Gastrodermis, iOo = oogonia, mOo = mature oocyte, SV = sperm vesicle, TL = testicular lobes, Vit = vitellarium. Means ± SD of 3 independent experiments (n = 3) with 4–6 worms per experiment are shown.

**Figure 7:**
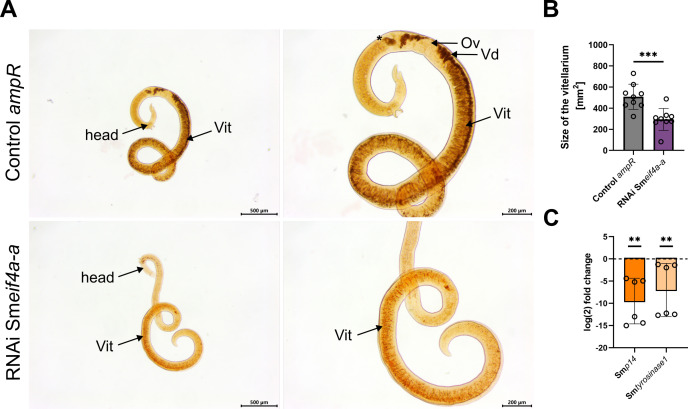
Sm*eif4a-a* RNAi impaired vitellogenesis in females. **(A)** Oil-red O-stained females of the RNAi group, which had been separated from couples before staining, showed weaker signals in the vitellarium and the vitelloduct compared to the control females incubated with *ampR* dsRNA (left, overviews; right, close-ups). As expected, no staining was found in the ovary, where no lipids are deposited (Kunz, 2001; Lu et al., 2015). **(B)** The area of the vitellarium was quantified and found to be significantly smaller in females of the Sm*eif4a-a* RNAi group. ***p < 0.001, determined by Mann-Whitney test. **(C)** RT-qPCRs exhibited significantly reduced transcript levels of the vitellarium marker genes Sm*p14* and Sm*tyrosinase1* after Sm*eif4a-a* RNAi. The housekeeping gene Sm*letm1* was used for normalization (Haeberlein et al., 2019). **p < 0.01, determined by *t*-test. Asterisk marks an egg in the ootype. Ov = ovary, Vd = vitelloduct, Vit = vitellarium. The asterisk marks an egg inside the ootype. **(A, B)**, means ± SD of 3 independent experiments (n = 3) with 3 worms per experiment are shown. **(C)**, means ± SD of 6 independent experiments (n = 6) are depicted.

To gain first insights into the potential functions of Sm*eif4a-a*, gene-specific KD experiments were performed by RNAi. The used dsRNAs had sizes of ~ 500 bp and were not covered by the RT-qPCR primers used for downstream analysis. The designed dsRNA was analyzed using the small interfering RNA-finder (si-fi) software to predict potential off-targets (Lück et al., 2019) as previously described (Moescheid et al., 2023). None of the listed off-targets with > 3 siRNA hits were annotated as RNA helicases, showing that the used dsRNA had a certain specificity for Sm*eif4a-a*. RNAi experiments were performed with 30 couples each and performed with six biological replicates. Couples incubated with a similar volume of DEPC-treated water, the solvent for dsRNA, or with non-schistosomal dsRNA (*ampR*) of the same concentration as in the RNAi group, served as controls. Previous studies showed no effects of *ampR* dsRNA on pairing stability, motility, egg production, and morphology of *S. mansoni* couples at concentrations between 30 – 60 µg/ml (Moescheid et al., 2023; Li et al., 2024). In this study, lower concentrations of dsRNA (15 µg/ml; 10 µg/ml) were applied, therefore a comparison between *ampR* dsRNA (15 µg/ml; 10 µg/ml) and a corresponding water control was performed to evaluate *ampR* as suitable dsRNA control at the concentrations used in this study. Although egg production was significantly higher in the *ampR* group compared to the water control (no dsRNA) at d 13 and d 15 of treatment, *ampR* dsRNA (15 µg/ml) showed no further effects on worm viability, morphology, or transcript levels of the genes examined in this study (**Supplementary Figure S6**). Therefore, the *ampR* dsRNA control was used in the following to interpret Sm*eif4a-a* RNAi effects. After 21 d of dsRNA treatment, the KD efficiency was determined. For this, couples were separated, and RNA from each gender was isolated and used for RT-qPCR. The results confirmed an efficient Sm*eif4a-a* KD of 88% in males and females, respectively (88.0 ± 1.0%, 88.0 ± 1.4% reduction compared to the water control; **Figure 5A**). The remaining protein level of SmeIF4A-a after RNAi could not be analyzed due to the lack of an appropriate antibody. The transcript level of isoform Sm*eif4a-b* was also analyzed, but it was unaffected upon Sm*eif4a-a* RNAi (**Supplementary Figure S7**). During the treatment period, control and Sm*eif4a-a* RNAi worms were monitored every 2–3 d and evaluated for physiological or morphological changes based on a standardized scoring system (Moescheid et al., 2023; Li et al., 2024). KD of Sm*eif4a-a* caused no pairing instability (**Figure 5B**), while worm attachment and motility were significantly reduced after 20 and 21 d of treatment, respectively (**Figures 5C, D**). Furthermore, eggs laid *in vitro* were quantified and categorized as abnormal or normal eggs depending on their size and shape. Examples for abnormal eggs are shown in **Supplementary Figure S8**. The overall egg production was evaluated by calculating abnormal plus normal eggs per couple. After 10 d of treatment, egg production was significantly reduced (**Figure 5E**) in the Sm*eif4a-a* RNAi group and further declined until no eggs were laid after 21 d (data not shown). The percentage of abnormal eggs did not significantly differ between Sm*eif4a-a* dsRNA-treated and control worms (**Figure 5F**), although a tendency of more abnormal eggs was found from d 10 on. Next, we investigated the morphology of the reproductive organs of all worm groups after 21 d of treatment by CLSM (**Figure 6**). Z-stacks of these organs were acquired to compare volumes (**Figure 6B**). This analysis revealed reduced volumes of the testicular lobes and the ovary in the Sm*eif4a-a* RNAi group (**Figures 6A, B**) compared to the control group. In contrast to control males, which possess densely filled testes, empty spaces were observed within the testicular lobes of Sm*eif4a-a* RNAi males. In ovaries of this group, no clear separation between both parts of the ovary was found, and low numbers of immature and mature oocytes appeared to be mixed within the comparatively small ovary. In addition, the total number of vitellocytes and the amount of stage 3/4 vitellocytes (Kunz, 2001; Lu et al., 2015) seemed to be reduced in the vitellarium of Sm*eif4a-a* RNAi females (**Figure 6A**). To substantiate this RNAi effect on vitellocytes, worms were incubated in Oil-red O to stain lipid droplets that are characteristic for fully differentiated S3/S4 cells (Kunz, 2001; Lu et al., 2015). Following Sm*eif4a-a* RNAi, the vitellarium exhibited lower staining intensities compared to the controls. Furthermore, in contrast to the control (*ampR* dsRNA), in which intensively stained S4 vitellocytes accumulated in the vitelloduct (which connects the vitellarium with the ootype) and thus made this structure evident, in females of the Sm*eif4a-a* RNAi group the vitelloduct was hardly visible (**Figure 7A**). This can be explained by the absence of Oil-red O-stained S3/S4 vitellocytes, which corresponds to the CLSM data. Next, we quantified the area of the vitellarium and found a significantly smaller size of this organ following Sm*eif4a-a* RNAi (**Figure 7B**). Additionally, we determined the relative transcript levels of the eggshell precursor protein p14 (Sm*p14*) and tyrosinase 1 (Sm*tyrosinase1*), as vitellarium marker genes (Ebersberger et al., 2005; Fitzpatrick et al., 2007). RT-qPCRs of control and Sm*eif4a-a* RNAi worms showed significantly lower transcript levels for both marker genes in females of the RNAi group (**Figure 7C**).

### 3.5 Sm*eif4a-a* RNAi reduced stem-cell proliferation

**Figure 8:**
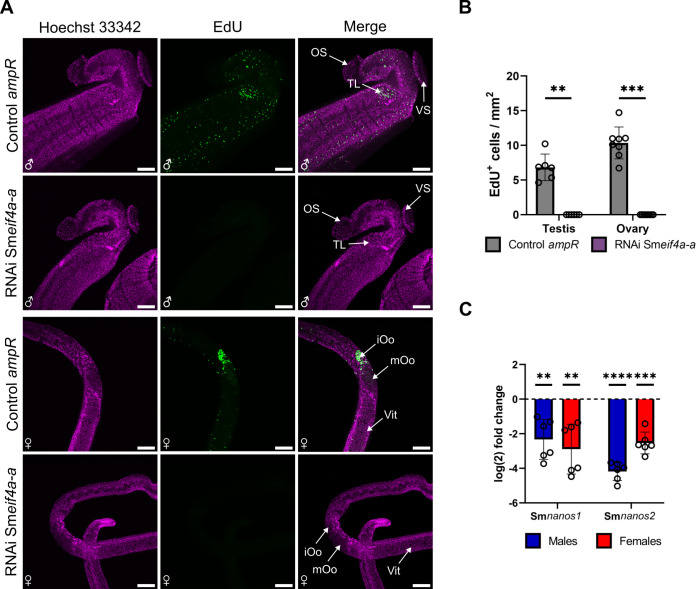
Sm*eif4a-a* RNAi led to a loss of stem-cell proliferation. **(A)** CLSM analysis of EdU-treated males and females after Sm*eif4a-a* RNAi revealed the absence of EdU signals (green) compared to the control (*ampR* dsRNA). Total DNA was stained by Hoechst 33342 (magenta). **(B)** The number of EdU^+^ cells was quantified and confirmed the absence of EdU^+^ cells in the testis and the ovary after Sm*eif4a-a* RNAi. **(C)** RT-qPCR revealed significantly reduced transcript levels of the stem-cell marker genes Sm*nanos1* and Smn*anos2* in males and females after Sm*eif4a-a* RNAi. The housekeeping gene Sm*letm1* was used for normalization (Haeberlein et al., 2019). **p < 0.01, ***p < 0.001, ****p < 0.0001, determined by *t*-test. Scale bars: 100 µm. iOo = oogonia, mOo = mature oocyte, OS = oral sucker, TL = testicular lobes, Vit = vitellarium, VS = ventral sucker. **(A, B)**, means ± SD of 3 independent experiments (n = 3) with 2–3 worms per experiment are shown. **(C)**, means ± SD of 6 independent experiments (n = 6) are depicted.

EdU assays were performed to comparatively investigate stem-cell proliferation in testes and ovaries of the treatment groups. No EdU^+^ cells were detected in males and females after Sm*eif4a-a* RNAi, whereas EdU^+^ cells occurred in the control with irrelevant dsRNA (*ampR*; **Figures 8A, B**). The absence of EdU^+^ cells was not restricted to the reproductive organs where GSCs are located (Wang et al., 2018; Lee, 2023). In addition, we observed no EdU^+^ cells in SSCs (neoblasts; Collins et al., 2013) around the gut, in the parenchyma, and next to the tegument. A similar phenotype was found before in a large-scale, unbiased RNAi study that aimed to identify therapeutic targets in *S. mansoni*. However, no causal link between SmeIF4A-a and its role in stem-cell proliferation was described (Wang et al., 2020). The results of EdU assays were substantiated by RT-qPCR results obtained for the stem cell marker genes Sm*nanos-1* and Sm*nanos-2*, which were shown before to regulate proliferation processes in GSCs and SSCs (Collins et al., 2013; Wang et al., 2013, Wang et al., 2018). Upon Sm*eif4a-a* RNAi, the transcript levels of both genes were significantly downregulated in males and females, which corresponds to the results of the EdU assays (**Figure 8C**).

### 3.6 No clear phenotype was observed after Sm*eif4a-b* RNAi

**Figure 9:**
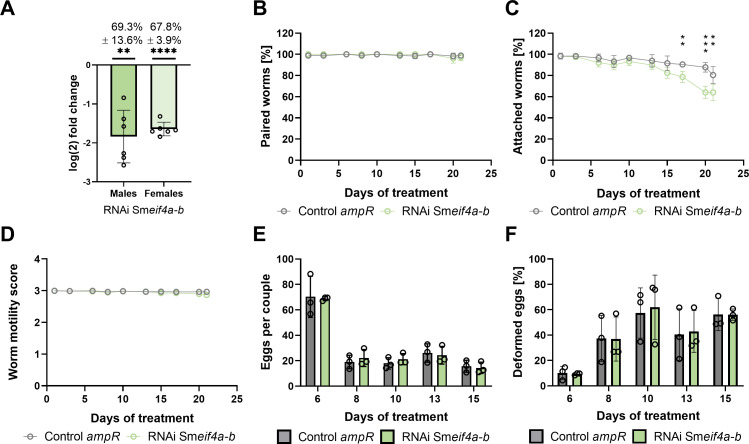
Sm*eif4a-b* RNAi reduced worm attachment. *S. mansoni* couples were treated with 10 µg/ml Sm*eif4a-b* dsRNA (green) every 2 to 3 d, or they were incubated with *ampR* dsRNA (10 µg/ml) as irrelevant dsRNA control (grey) over a period of 21 d **(A)** Knockdown efficiency was determined by RT-qPCR measuring relative transcript levels in males and females after separation of the couples. The housekeeping gene Sm*letm1* was used for normalization (Haeberlein et al., 2019). The following phenotypes were found in the Sm*eif4a-a* RNAi group: **(B)** pairing stability was constant, **(C)** worm attachment significantly decreased after 17 d, **(D)** worm motility, scored from 0 (no motility) to 4 (hyperactivity), was constant, **(E)** total egg production and the ratio of abnormal eggs **(F)** was similar to that of the control. **p < 0.01, ***p < 0.001, ****p < 0.0001, determined by *t*-test. A-D, n = 6; E-F, n = 3.

**Figure 10:**
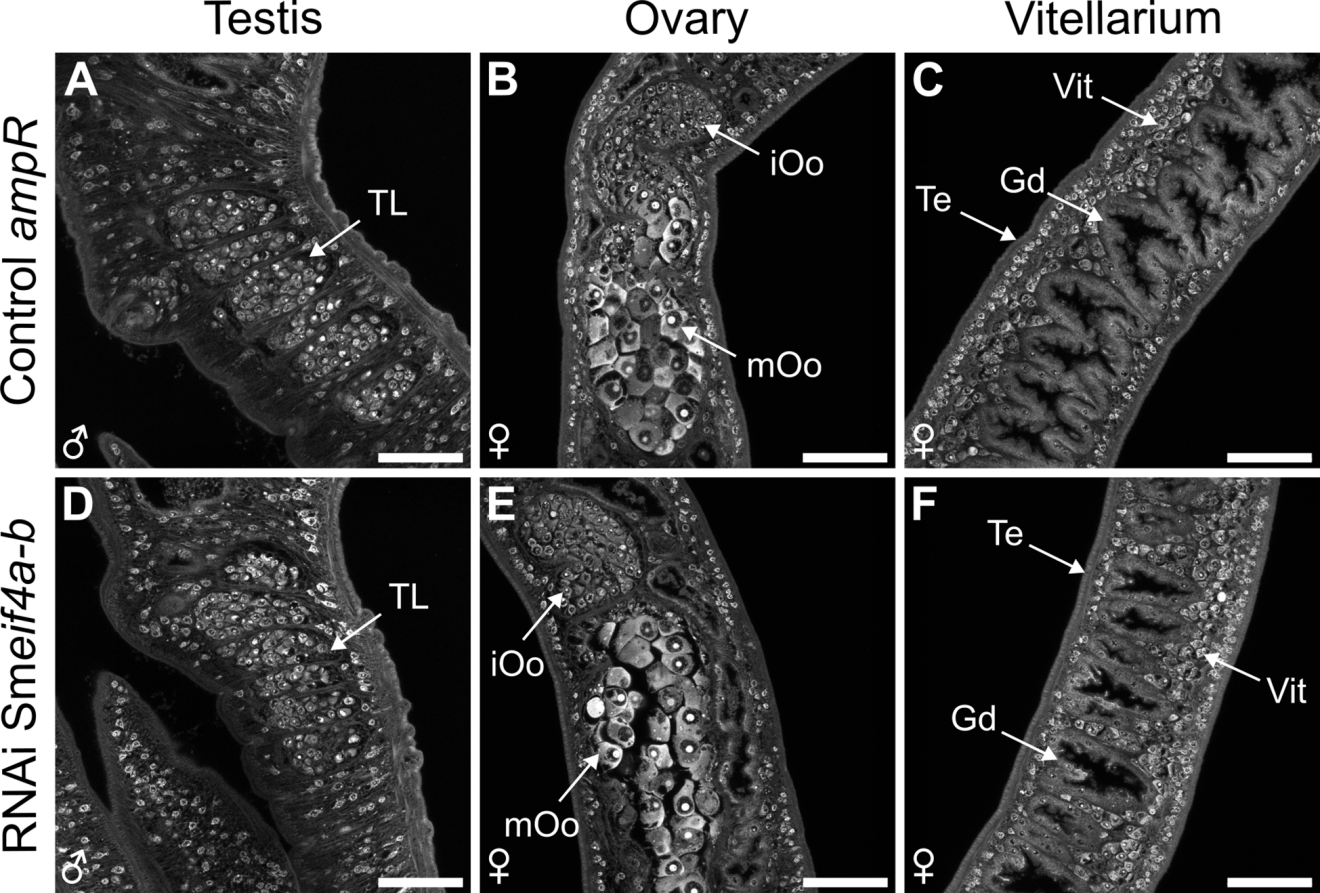
Sm*eif4a-b* RNAi caused no morphological phenotypes in the reproductive organs. CLSM analysis of carmine-red stained males and females after separation of the couples showed no differences between the Sm*eif4a-b* RNAi group and the control (*ampR* dsRNA). Scale bars: 50 µm. Gd = Gastrodermis, iOo = oogonia, mOo = mature oocyte, Te = tegument, TL = testicular lobes, Vit = vitellarium. Representative images of 3 independent experiments (n = 3) with 4–6 worms per experiment are shown.

**Figure 11:**
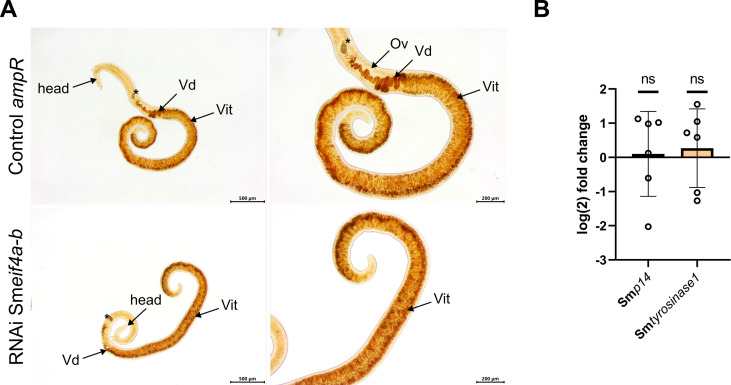
Sm*eif4a-b* RNAi showed no effects on vitellogenesis in females. **(A)** Following Sm*eif4a-b* RNAi, Oil-red O-stained females, which were separated from males for analysis, showed staining intensities comparable to control females (*ampR* dsRNA). **(B)** RT-qPCR of the vitellarium marker genes Sm*p14* and Sm*tyrosinase1* showed no difference between the Sm*eif4a-b* RNAi group and the control. The housekeeping gene Sm*letm1* was used for normalization (Haeberlein et al., 2019). Ov = ovary, Vd = vitelloduct, Vit = vitellarium. Asterisk marks an egg in the ootype. **(A)**, representative images of 3 independent experiments (n = 3) with 3 worms per experiment are shown. **(B)**, means ± SD of 6 independent experiments (n = 6) are depicted.

**Figure 12:**
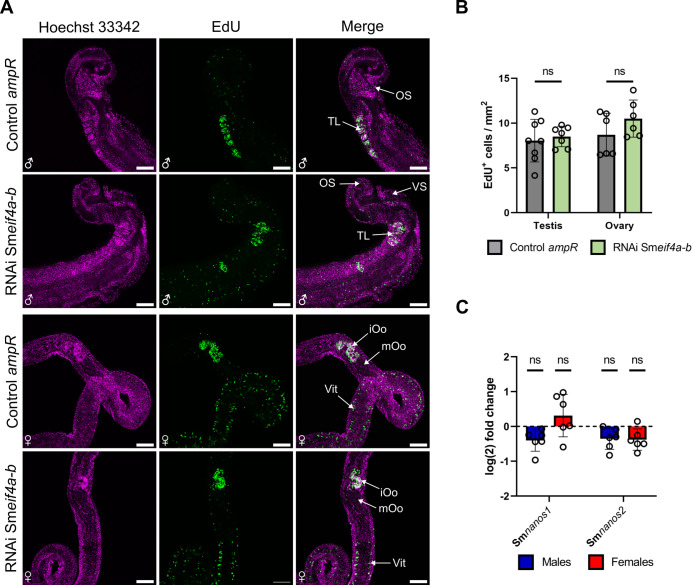
Sm*eif4a-b* RNAi showed no effects on stem-cell proliferation. **(A)** CLSM analysis of EdU-treated males and females after Sm*eif4a-b* RNAi showed similar EdU signals (green) compared to the control (*ampR* dsRNA). Total DNA was stained by Hoechst 33342 (magenta). **(B)** The number of EdU^+^ cells was quantified in the testis and the ovary and revealed no difference between the Sm*eif4a-b* RNAi group and the control. **(C)** Relative transcript levels of the stem-cell marker genes Sm*nanos1* and Smn*anos2*, as analyzed by RT-qPCR, remained similar between the Sm*eif4a-b* RNAi group and the control. The housekeeping gene Sm*letm1* was used for normalization (Haeberlein et al., 2019). Scale bars: 100 µm. iOo = oogonia, mOo = mature oocyte, OS = oral sucker, TL = testicular lobes, Vit = vitellarium, VS = ventral sucker. A-B, means ± SD of 3 independent experiments (n = 3) with 2–3 worms per experiment are shown. **(C)**, means ± SD of 6 independent experiments (n = 6) are depicted.

As for Sm*eif4a-a*, RNAi experiments were performed to functionally characterize Sm*eif4a-b* in male and female worms. To this end, Sm*eif4a-b* dsRNA was administered to worm couples every 2–3 d, which resulted in KD efficiencies of 69.3 ± 13.6% and 67.8 ± 3.9% in males and females, respectively, after 21 d of treatment (**Figure 9A**). Analysis of potential off-targets by si-fi (Lück et al., 2019) predicted four genes with > 3 siRNA hits. These genes were annotated as RNA helicases including Sm*eif4a-a* (Smp_097660). RT-qPCR showed lower transcript levels for three of these genes, Smp_333940, Smp_034190, and Smp_031010, but only in males and not in females (**Supplementary Figure S9**). Transcript levels of Sm*eif4a-a* were unaffected by the KD of Sm*eif4a-b* in males and females, indicating no reciprocal influence of RNAi on the transcript levels of each of these genes. During the treatment period of 21 d, the worms were examined for morphological or physiological phenotypes – as for Sm*eif4a-a*. While pairing stability, motility, total egg production, and the percentage of abnormal eggs were similar between the Sm*eif4a-b* RNAi group and the control (*ampR* dsRNA), only worm attachment was significantly reduced after 17 d of Sm*eif4a-b* RNAi (**Figure 9**). After 21 d, only about 60% of the worms were still attached (**Figure 9C**). The morphology of the reproductive organs of both genders and stem-cell proliferation of GSCs and SSCs were evaluated in control and Sm*eif4a-b* RNAi worms by CLSM, lipid staining, and EdU assays. CLSM analysis of the reproductive organs of testis, ovary, and vitellarium, revealed no differences between the control and the Sm*eif4a-b* RNAi group (**Figure 10**). The vitellarium was studied in more detail by staining the lipid droplets of mature vitellocytes and quantifying the relative transcript levels of Sm*p14* and Sm*tyrosinase1* as marker genes. Lipid staining of control and Sm*eif4a-b* RNAi worms resulted in similar staining intensities between both treatment groups (**Figure 11A**). Furthermore, the relative transcript levels of Sm*p14* and Sm*tyrosinase1* were not significantly different from those of the control group (**Figure 11B**). Next, EdU assays to investigate stem-cell proliferation of GSCs and SSCs revealed no differences of the numbers of EdU^+^ cells in ovaries and testes upon Sm*eif4a-b* RNAi (**Figures 12A, B**). In males and females, finally, the transcript levels of Sm*nanos1* and Sm*nanos2* as stem-cell marker genes were similar to those of the control (**Figure 12C**).

A comparison between the water control and 10 µg/ml *ampR* dsRNA was performed for each type of post-RNAi analysis to confirm the suitability of *ampR* dsRNA as irrelevant dsRNA control. The only difference between the water and *ampR* group was found on d 13 with a significantly higher egg production of *ampR* worms. Otherwise, *ampR* dsRNA (10 µg/ml) had no effects on worm viability, morphology, stem-cell proliferation or transcript levels of the genes examined in this study (**Supplementary Figure S10**).

## 4 Discussion

Schistosomes have a unique reproductive biology that is not yet completely understood. Part of the developmental program is organized by stem cells (Wang et al., 2018), and part of the molecular program to initiate and maintain the pairing-dependent differentiation of female gonads may include the activity of RNA helicases. Among other important functions, these enzymes are involved in germline development as shown in model organisms. In *Caenorhabditis elegans*, DEAD-box helicases occur in germinal granules, in mice they are associated with perinuclear granules in testis germ cells that may represent nuage, and in *Drosophila* and *Xenopus*, they are also expressed in germ cells and control germline development by translation regulation (Saffman and Lasko, 1999). One of the 33 DEAD-box RNA helicases in *S. mansoni*, Smv*lg1*, was already characterized. Sm*vlg1* RNAi reduced the volume of female ovaries and thus appeared to be involved in oogenesis (Skinner et al., 2020). To uncover further functions of DEAD-box RNA helicases in *S. mansoni*, we focused on eIF4A, orthologs of which are involved in translation initiation (Rozen et al., 1990). Similar to model organisms, three eIF4A isoforms were found in platyhelminth species, one of them was identified as eIF4AIII due to additional conserved motifs (Li et al., 1999). Surprisingly, the high sequence similarities between eIF4AI and eIF4AII found in model organisms (Nielsen and Trachsel, 1988), was not confirmed for eIF4A-a and eIF4A-b of platyhelminths, which may suggest less redundant roles of both isoforms in this phylum. In human and mouse, *eif4a1* is the dominantly transcribed isoform, but the ratio of *eif4a1* to *eif4a2* transcripts depends on the tissue type (Nielsen and Trachsel, 1988; Galicia-Vázquez et al., 2012). While *eif4a1* levels dominate during growth, *eif4a2* levels increase upon its arrest demonstrating a growth-dependent isoform switching (Williams-Hill et al., 1997). Similar findings were observed in *S. mansoni*. Bulk RNA-seq data showed overall higher transcript levels of Sm*eif4a-a* compared to Sm*eif4a-b* in males and females, as well as in their isolated gonads, regardless of their pairing status (Lu et al., 2016). Based on scRNA-seq data, Sm*eif4a-a* is highly transcribed in various tissues, but its transcript level dominates in ovaries of bF (Lu et al., 2016; Wendt et al., 2020), especially in late germ cells as determined by bulk scRNA-seq of isolated ovaries (Moescheid et al., 2025). This corresponds to our WISH results that localized Sme*if4a-a* transcripts predominantly in the mature part of the ovary.

ScRNA-seq data suggested that Sm*eif4a-b* transcripts occur in somatic cells such as muscles and neurons, as well as in male gametes and GSC progeny (Wendt et al., 2020). Our RT-qPCR analysis revealed a slightly higher transcript level of Sm*eif4a-a* in bF versus sF, while significantly lower transcript levels of Sm*eif4a-b* were found in bF compared to sF. Since many growth and proliferation processes are initiated in females upon pairing (Kunz, 2001; Lu et al., 2016; Popiel and Basch, 1984), the pairing-influenced bias of the transcript levels of Sm*eif4a-a* and Sm*eif4a-b* between bF and sF seems plausible. In males, however, pairing appeared to have no influence on the transcript levels of both genes. Although SmeIF4A-a and SmeIF4A-b are more distinct to each other than the two human eIF4A paralogs, the differences in expression levels and transcript localization between Sm*eif4a-a* and Sm*eif4a-b* show parallels to Hs*eif4a1* and Hs*eif4a2*.

Functional analysis of Sm*eif4a-a* by RNAi uncovered its role in stem-cell proliferation, maintenance of reproductive organs, and egg production. These results are consistent with findings from model organisms. In humans, eIF4AI was shown to be required for cellular proliferation, vitality, and protein synthesis (Galicia-Vázquez et al., 2012). As an RNA helicase, eIF4A is involved in translation initiation of selected mRNAs, thereby regulating the level of protein expression post-transcriptionally (Galicia-Vázquez et al., 2012). Ribosome profiling studies identified cell cycle regulators and proto-oncogenes being eIF4A dependent (Rubio et al., 2014; Wolfe et al., 2014; Modelska et al., 2015). The identified cell cycle regulators included cyclin D1, cyclin D2, and the cyclin-dependent kinase 6 (CDK6), which promote the progression from the G1- to the S-phase (Zhang and Li, 2021). Similar cell cycle regulators involved in the G1/S transition might be eIF4A-a-dependent in *S. mansoni*, as EdU is incorporated into DNA during the S-phase, and EdU^+^ signals were absent after Sm*eif4a-a* RNAi. Furthermore, an impaired cell cycle progression and thus a loss of GSCs after Sm*eif4a-a* RNAi might explain the reduced transcript levels of the stem-cell marker genes Sm*nanos-1* and Sm*nanos-2*. It has been shown that *nanos-1* is involved in the maintenance of GSCs (Wang et al., 2018, Wang et al., 2007; Wang and Lin, 2004) and *nanos-2* is important for proliferation and maintenance of SSCs (Collins et al., 2013; Wang et al., 2018, Wang et al., 2013). Previous studies demonstrated that Sm*nanos-1* RNAi in mature females led to a reduction in ovary volume and stem-cell proliferation (Moescheid et al., 2025). In the free-living planarian *S. mediterranea, nanos* RNAi resulted in the loss of the testis (Wang et al., 2007). Nanos acts as a translational repressor to prevent GSCs from entering oogenesis and spermatogenesis precociously (Kraemer et al., 1999; Wang and Lin, 2004; Lai et al., 2010). In our study, the reduced Sm*nanos-1* level probably explains why mature oocytes, which had already entered oogenesis, were detected in the immature part of the ovary. Moreover, the decreased Sm*nanos-1* level potentially prevented the self-renewal of GSCs, leading to the observed reduced cell numbers in testes, ovaries, and vitellaria following Sm*eif4a-a* RNAi. This explains the smaller dimensions of the reproductive organs following Sm*eif4a-a* RNAi and empty spaces in these organs. Impaired vitellogenesis, finally, explains the reduced egg production, a finding substantiated by the analysis of the transcript levels of genes such as Sm*p14* and Sm*tyrosinase1* that were shown before to be essential for egg production in *S. mansoni* (DeWalick et al., 2012; Fitzpatrick et al., 2007). To uncover the mRNAs that depend on the unwinding activity of SmeIF4A-a will be a topic of future studies.

We compared all post-RNAi analyses between the *ampR* dsRNA (10 µg/ml and 15 µg/ml) and the water (no dsRNA) control to verify the suitability of this non-helminth dsRNA also at lower concentrations compared to previous studies (Moescheid et al., 2023, Moescheid et al., 2025; Li et al., 2024). EdU assays of worms treated with *ampR* dsRNA and without dsRNA (DEPC-water control) revealed no influence of this irrelevant dsRNA on the proliferation of SSCs and GSCs in the testis and the ovary. Furthermore, vitellogenesis was unaffected by *ampR* dsRNA as demonstrated by comparable lipid-staining intensities of females of both groups. However, a higher egg production on d 13 (10 µg/ml *ampR*) or until the end of the experiment (15 µg/ml *ampR*) was found in the *ampR* group. Our results cannot be compared with earlier data from Li et al., 2024; Moescheid et al., 2025, Moescheid et al., 2023, as we used a different culture medium, and the effects on egg production had previously only been investigated up to d 10. For our RNAi approach, we used the improved culture medium ABC169/LDL (Wang et al., 2019; Li et al., 2023), which maintains female reproductive organs and thus egg production over a longer period of time. The influence of *ampR* dsRNA on later time points of egg production *in vitro* remains unknown.

For Sm*eif4a-b*, except reduced attachment of worms at d 17 of the *in vitro* culture, but earlier than for Sm*eif4a-a* worms (d 20), no clear effects were uncovered by RNAi. According to scRNA-seq data, Sm*eif4a-b* expression is comparatively high in muscles and neurons (**Supplementary Figure S4**), also compared to Sm*eif4a-a*, which may explain this observation and the later detachment effect following Sm*eif4a-a* RNAi. However, RT-qPCR analyses of four other RNA helicases annotated as potential off-targets exhibited reduced transcript levels for three predicted genes in males. One of these is SmeIF4AIII, which was shown before to induce detachment of *S. mansoni* after RNAi (Wang et al., 2020). Therefore, at this stage of the analysis, it is unclear whether the observed detachment is caused by reduced levels of Sm*eif4a-b*, or Sm*eif4a3*, or a combination of both. In humans, RNAi of Hs*eif4a2* failed to trigger a phenotype, while KD of Hs*eif4a1* reduced protein synthesis and blocked cell proliferation. Although HseIF4AII was upregulated after KD of Hs*eif4a1*, the phenotype could not be rescued, indicating that HseIF4AII is unable to fully compensate for HseIF4AI (Galicia-Vázquez et al., 2012). No upregulation of Sm*eif4a-b* was observed in *S. mansoni* after Sm*eif4a-a* RNAi or vice versa.

The absence of further phenotypic effects after Sm*eif4a-b* RNAi might have different reasons. KD efficiencies of about 70% may have been insufficient to induce clearer phenotypes. Alternatively, Sm*eif4a-b* might not be essential for the cell proliferation and reproduction of adult worms, which is supported by the lower transcript levels of this isoform compared to Sm*eif4a-a*. Bulk RNA-seq data indicated that Sm*eif4a-a* may represent the dominantly transcribed isoform with > 30x higher transcript levels in males and testis than Sm*eif4a-b* (**Supplementary Table S5**, Lu et al., 2016). In females, expression of Sm*eif4a-a* was about > 70x higher than Sm*eif4a-b*, and in ovaries of females this ratio even increased to approximately 100x (sO) and 330x (bO). Bulk RNA-seq data showed highest transcript levels of Sm*eif4a-b* in schistosomula (Lu et al., 2018), the first intra-mammalian stage. This suggests that SmeIF4A-b may play a more important role in this developing stage than in adult worms.

In summary, we have functionally characterized two helicase genes of *S. mansoni* and identified SmeIF4A-a as one factor that is involved in the unique reproductive biology of this parasite. As an RNA helicase, SmeIF4A-a fulfills important functions for stem-cell activity of GSCs and SSCs and differentiation processes in male and female gonads. Thus, our study contributes to the concept of schistosomiasis as a stem cell-governed disease with GSCs warranting the success of the life cycle by maintaining infectivity, and with SSCs promoting parasite longevity and survival in the host (Wendt and Collins, 2016). SmeIF4A-a is part of both stem-cell groups and as such of potential importance for stem-cell activity in general. In contrast, SmeIF4A-b exhibits no association with stem-cell activity and differentiation, but it appears to be more involved in processes controlling the attachment capacity of adult worms. Although RNAi showed no immediate vitality phenotype for both genes, against the importance of stem cells for schistosome biology, targeting SmeIF4A-a by inhibitors could open new perspectives for intervention strategies aiming to reduce egg-associated pathogenesis and to interrupt the life cycle by blocking parasite reproduction.

## Data Availability

The original contributions presented in the study are included in the article/**Supplementary Material**. Further inquiries can be directed to the corresponding authors.
