# Supplementary material for: The DEAD-box RNA helicase eIF4A is a crucial factor for stem-cell activity and reproduction of the parasite Schistosoma mansoni
Source: Front Cell Infect Microbiol. 2026 Jan 9;15:1731808. doi: 10.3389/fcimb.2025.1731808 (PMC12827517; doi:10.3389/fcimb.2025.1731808)
Supplement: Supplementary file 1 [file DataSheet1.pdf]

Supplementary Material

Supplementary Figures (S1 – S10; pages 1-8) and Supplementary Tables (S1 – S5, pages 9-10).

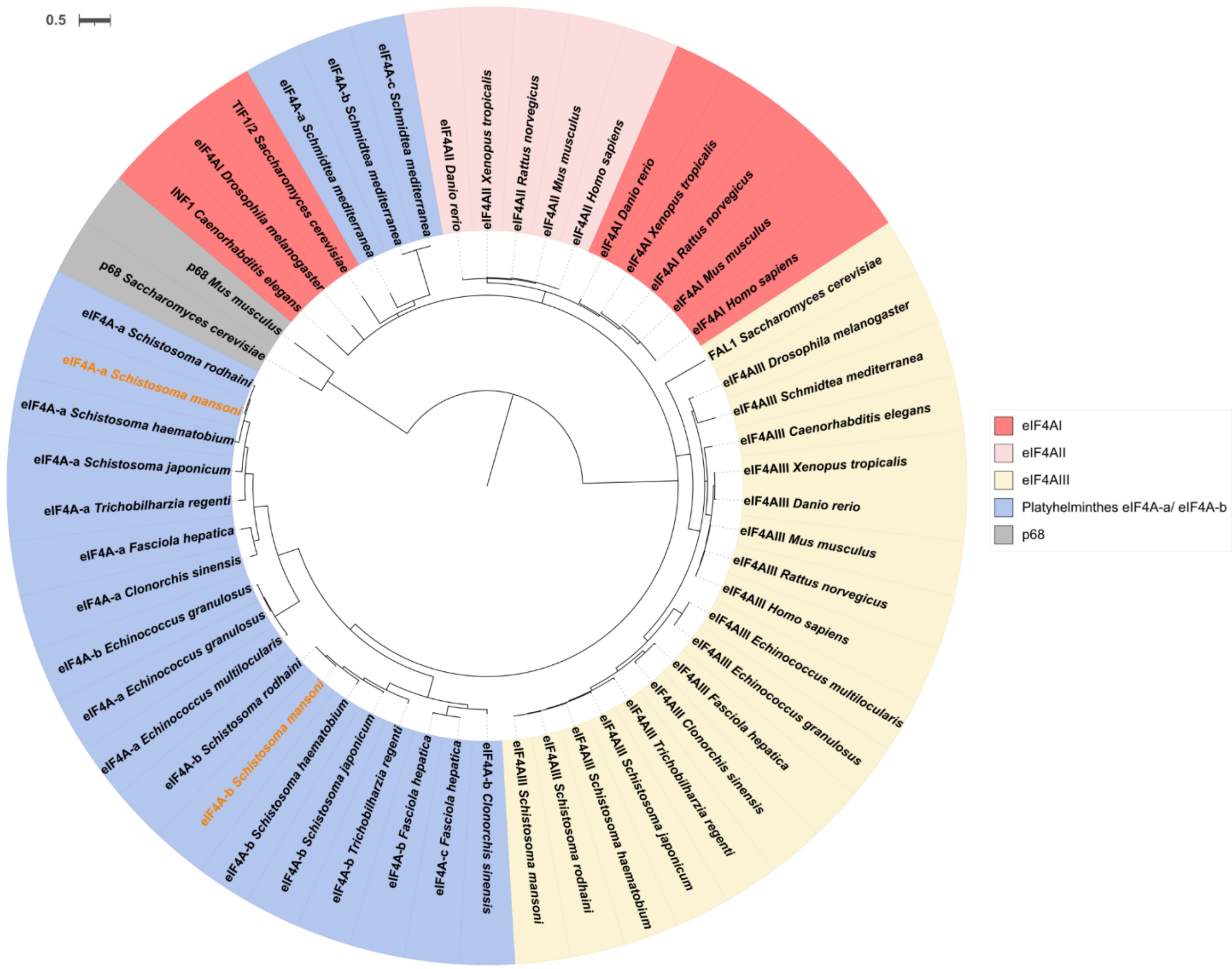

Supplemental Figure S1: Phylogenetic tree of eIF4A isoforms from model organisms and platyhelminths. The tree was generated using MrBayes (Ronquist et al., 2012). The amino acid sequence of the DEAD-box RNA helicases p68 from *Mus musculus* and *Saccharomyces cerevisiae* were used to root the tree. Branch lengths are indicated.

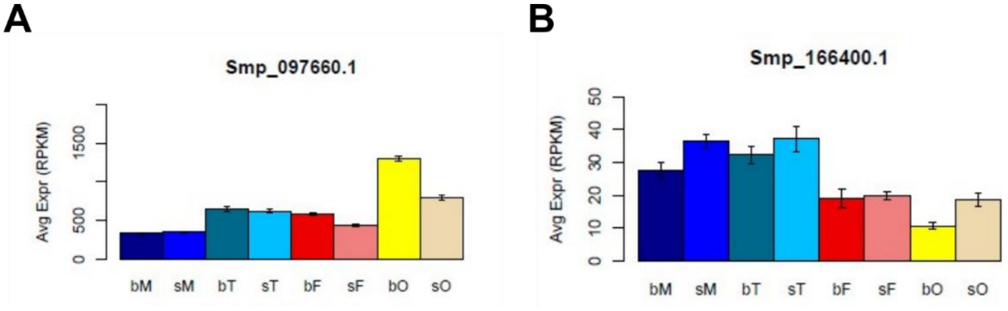

Supplemental Figure S2: Transcript levels of Sm*eif4a-a* **(A)** and Sm*eif4a-b* **(B)** determined by RNA-seq (Lu et al., 2016). The average expression level of each gene is shown for pairing-experienced (bM, bF) and pairing-unexperienced (sM, sF) males and females and their isolated gonads: testis (bT, sT) and ovary (bO, sO). RPKM = reads per kilobase million.

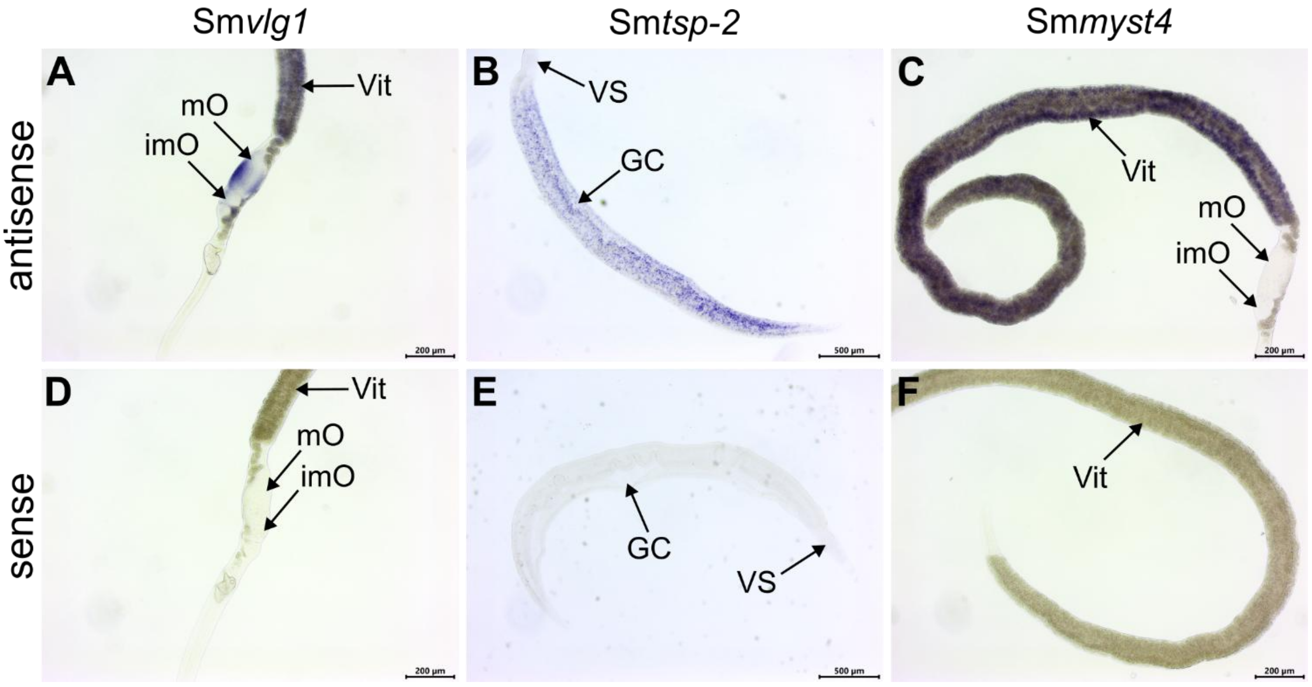

Supplemental Figure S3: WISH of already published riboprobes were used as methodological control. **(A-C)** Transcripts were localized using a gene-specific antisense riboprobe, while the corresponding sense probe served as control **(D-F)**. Sm*vlg1* (Skinner et al., 2012), Sm*tsp-2* (Cogswell et al., 2011), Sm*myst4* (Li et al., 2024). GC = gynaecophoric canal, imO = immature ovary, mO = mature ovary, Vit = vitellarium, VS = ventral sucker.

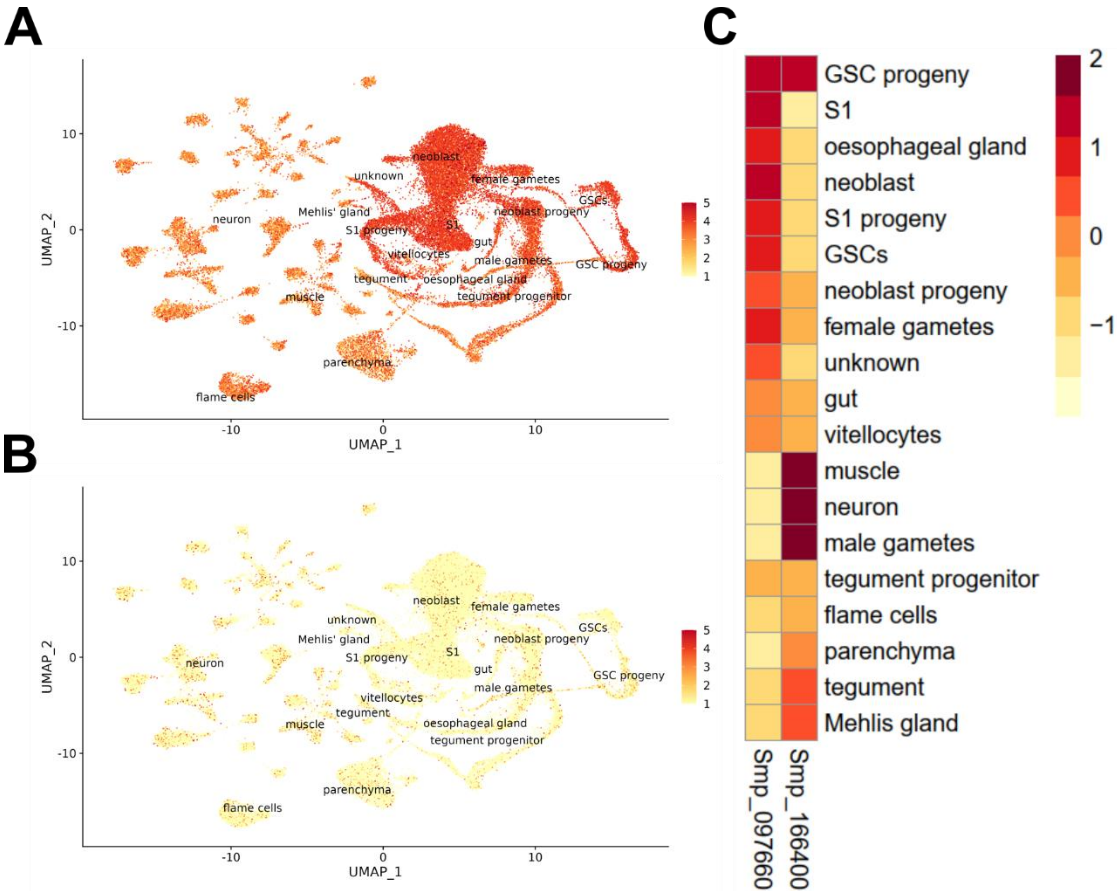

Supplemental Figure S4: UMAP plots of Sm*eif4a-a* and Sm*eif4a-b* in adult *S. mansoni* revealed by scRNA-seq (Wendt et al., 2020). **(A)** UMAP plot of Sm*eif4a-a* and **(B)** Sm*eif4a-b*. **(C)** Heatmap showing different expression levels of both Sm*eif4a* isoforms in the listed tissues. Colored by gene expression from yellow = low to dark red = high.

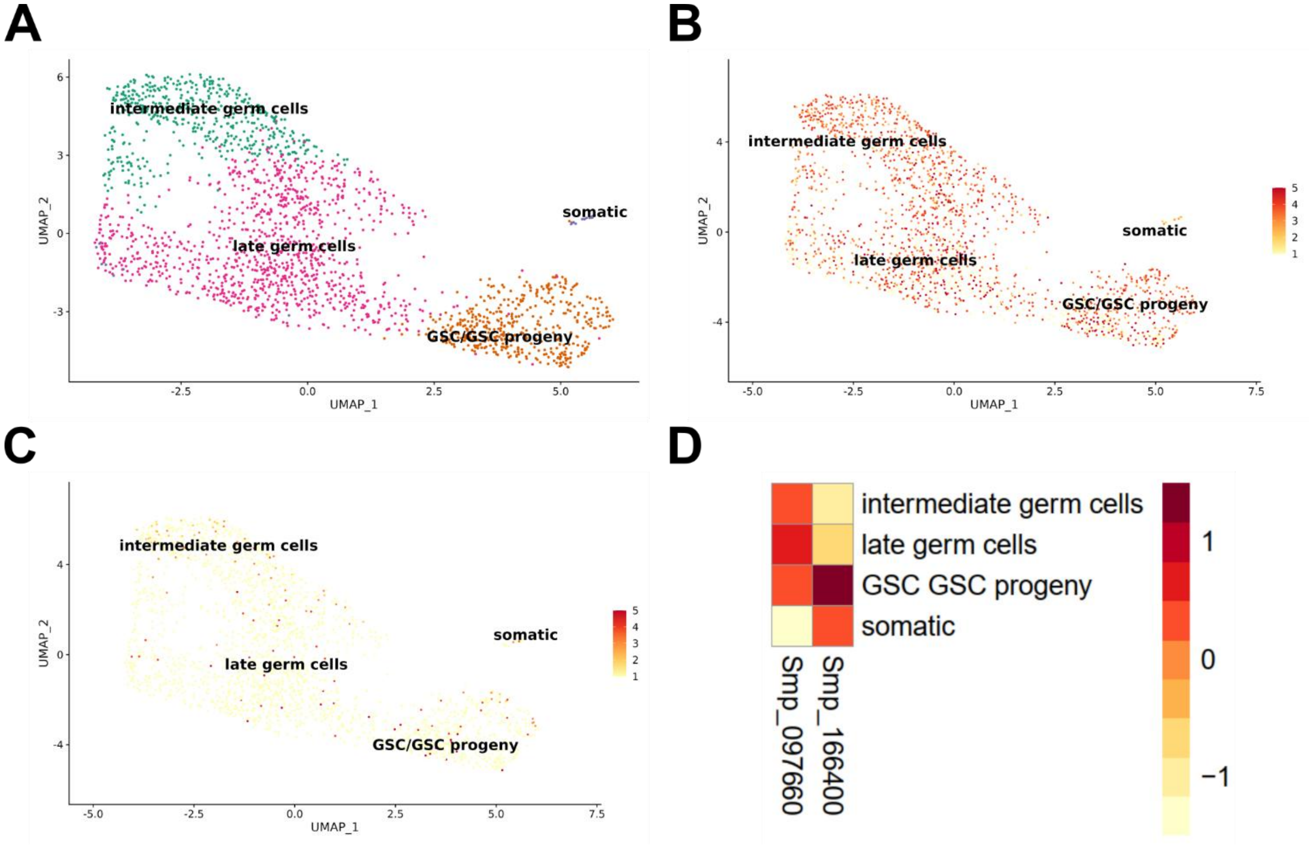

Supplemental Figure S5: ScRNA-seq data of Sm*eif4a-a* and Sm*eif4a-b* in isolated ovaries of mature females (Moescheid et al., 2025). **(A)** Uniform Manifold Approximation and Projection (UMAP) plot of the four cell clusters of isolated mature ovaries. **(B)** UMAP plot of Sm*eif4a-a*. **(C)** UMAP plot of Sm*eif4a-b*. **(D)** Heatmap of both Sm*eif4a* isoforms and their expression in the four cell clusters. UMAP plots and the heat map are colored by gene expression (yellow = low, dark red = high).

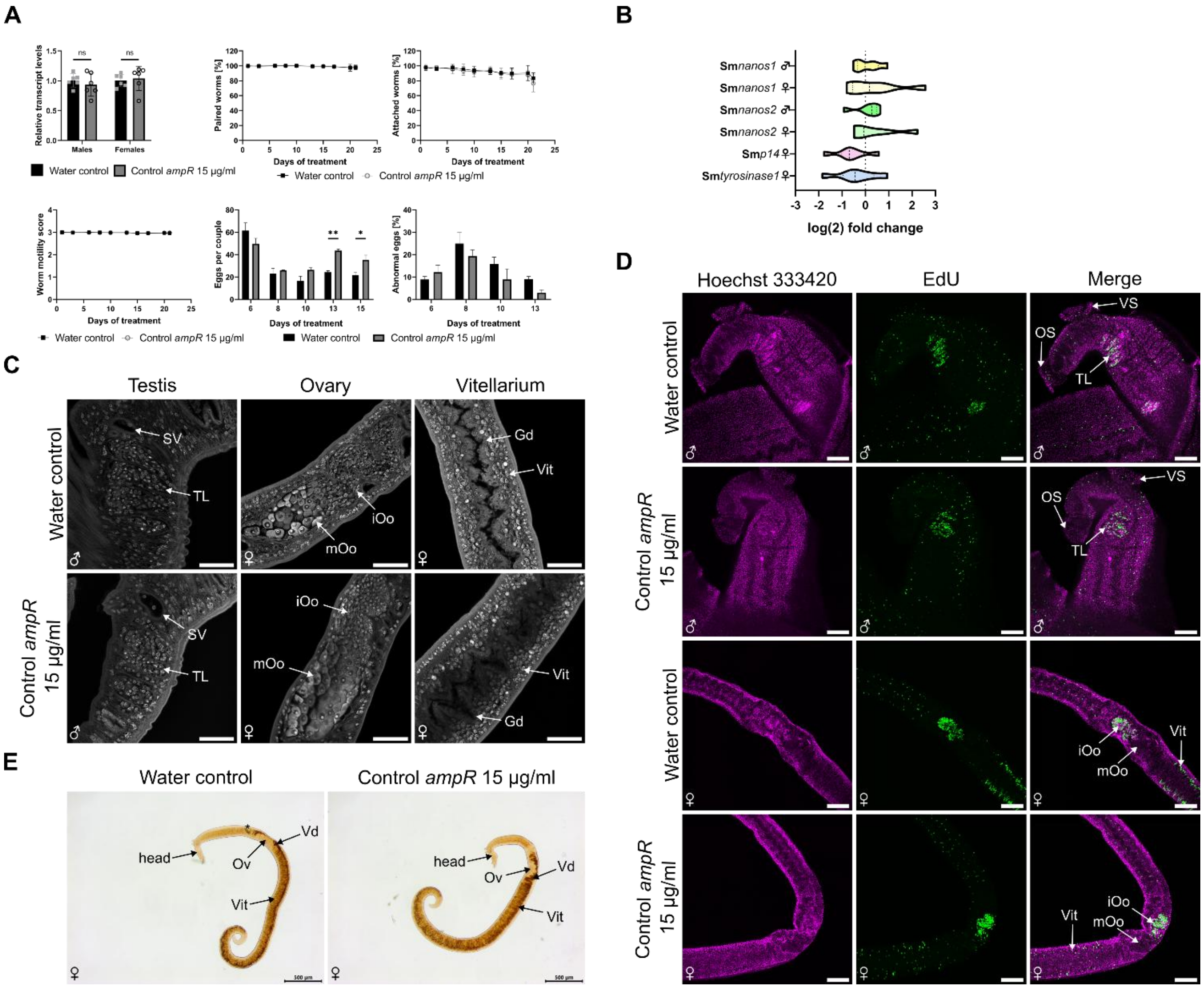

Supplemental Figure S6: Non-schistosomal dsRNA (*ampR*) at 15 µg/ml showed no effects on worm viability, morphology, and stem-cell proliferation. **(A)** Transcript levels of Sm*eif4a-a* at d 21, and the scoring parameters pairing stability, attachment, and motility were unaffected by addition of *ampR* dsRNA. Egg production was significantly higher 13 and 15 d after treatment, while the ratio of abnormal to normal eggs did not differ compared to the water control (no dsRNA). * $p < 0.05$, ** $p < 0.01$, determined by *t*-test. Except for egg numbers (n = 3), remaining data are n = 6. **(B)** Transcript levels of investigated genes were normalized using the housekeeping gene Sm*letm1* (Haeberlein et al., 2019), and were similar to the water control. **(C)** CLSM analysis showed normal morphologies of the reproductive organs of males and females. **(D)** EdU assays revealed comparable stem-cell proliferation in GSCs and SSCs between the *ampR* and water control. **(E)** Lipid staining of female *S. mansoni* was unaffected by incubation with *ampR* dsRNA. Scale bars: C, 50 µm; D, 100 µm. Gd = Gastrodermis, iOo = oogonia, mOo = mature oocyte, Ov = Ovary, SV = sperm vesicle, TL = testicular lobes, Vd = vitelloduct, Vit = vitellarium. B, means ± SD of 6 independent experiments (n = 6) are shown. C-E, representative images of 3 independent experiments (n = 3) with 3 (D, E) or 6 (C) worms per experiment, analyzed after 21 d of dsRNA treatment, are depicted.

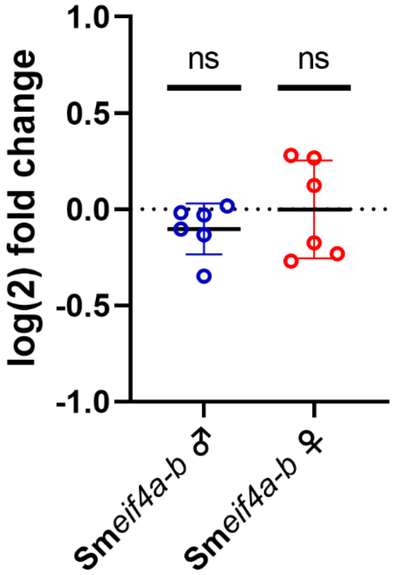

Supplemental Figure S7: The transcript level of Sm*eif4a-b* was unaffected after Sm*eif4a-a* RNAi. Transcript levels were quantified by RT-qPCR from cDNA of males (blue) and females (red), and were normalized using the housekeeping gene Sm*letm1* (Haeberlein et al., 2019); n = 6.

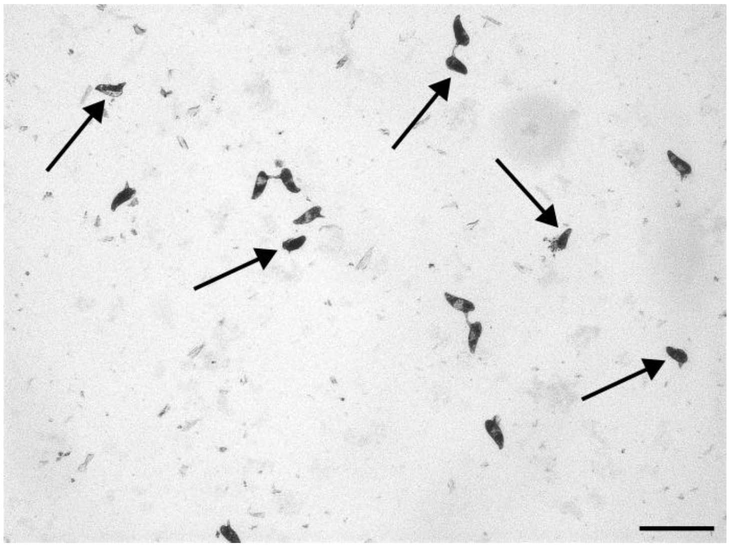

Supplemental Figure S8: Examples of abnormal *in vitro* laid eggs of *S. mansoni*. Eggs laid *in vitro* were classified as abnormal (arrows) when they were smaller, had an abnormal shape, or lacked the spine. Scale bar: 200 µm.

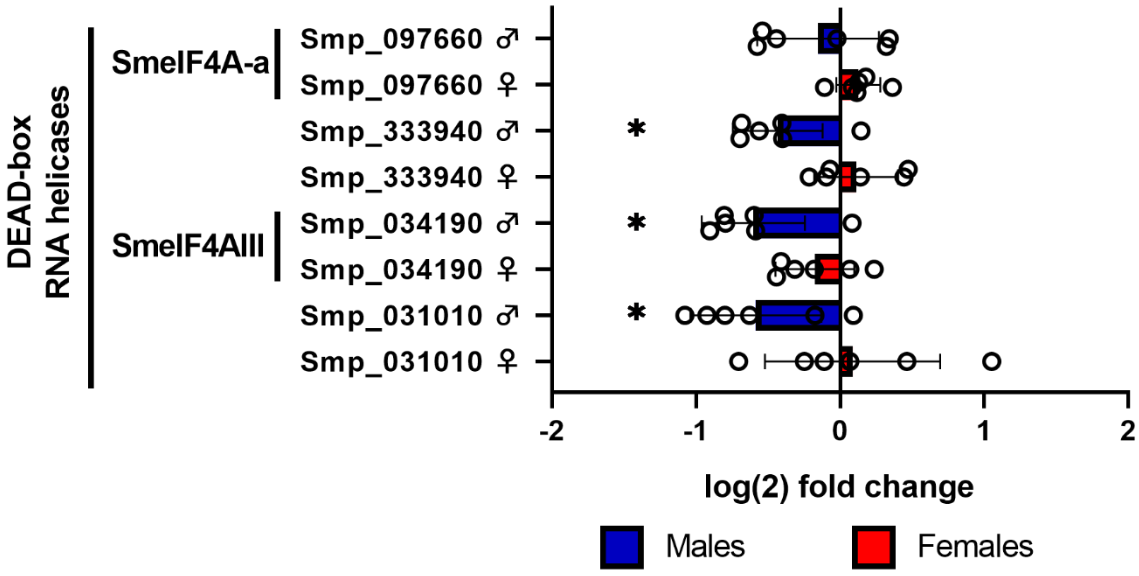

Supplemental Figure S9: RNAi of Sm*eif4a-b* resulted in decreased transcript levels of three off-target genes in male worms. Off-targets of Sm*eif4a-b* dsRNA were predicted using the si-fi software (Lück et al., 2019). In total, four DEAD-box RNA helicases were listed among the off-targets and their transcript levels relative to control worms (treated with *ampR* dsRNA) were determined by RT-qPCR in males (blue) and females (red) after 21 d of RNAi. The housekeeping gene Sm*letm1* was used for normalization (Haeberlein et al., 2019). Transcript levels of Sm*eif4a-a* were unaffected showing that the obtained results after RNAi of Sm*eif4a-b* are independent of Sm*eif4a-a*. * $p < 0.05$, determined by *t*-test.

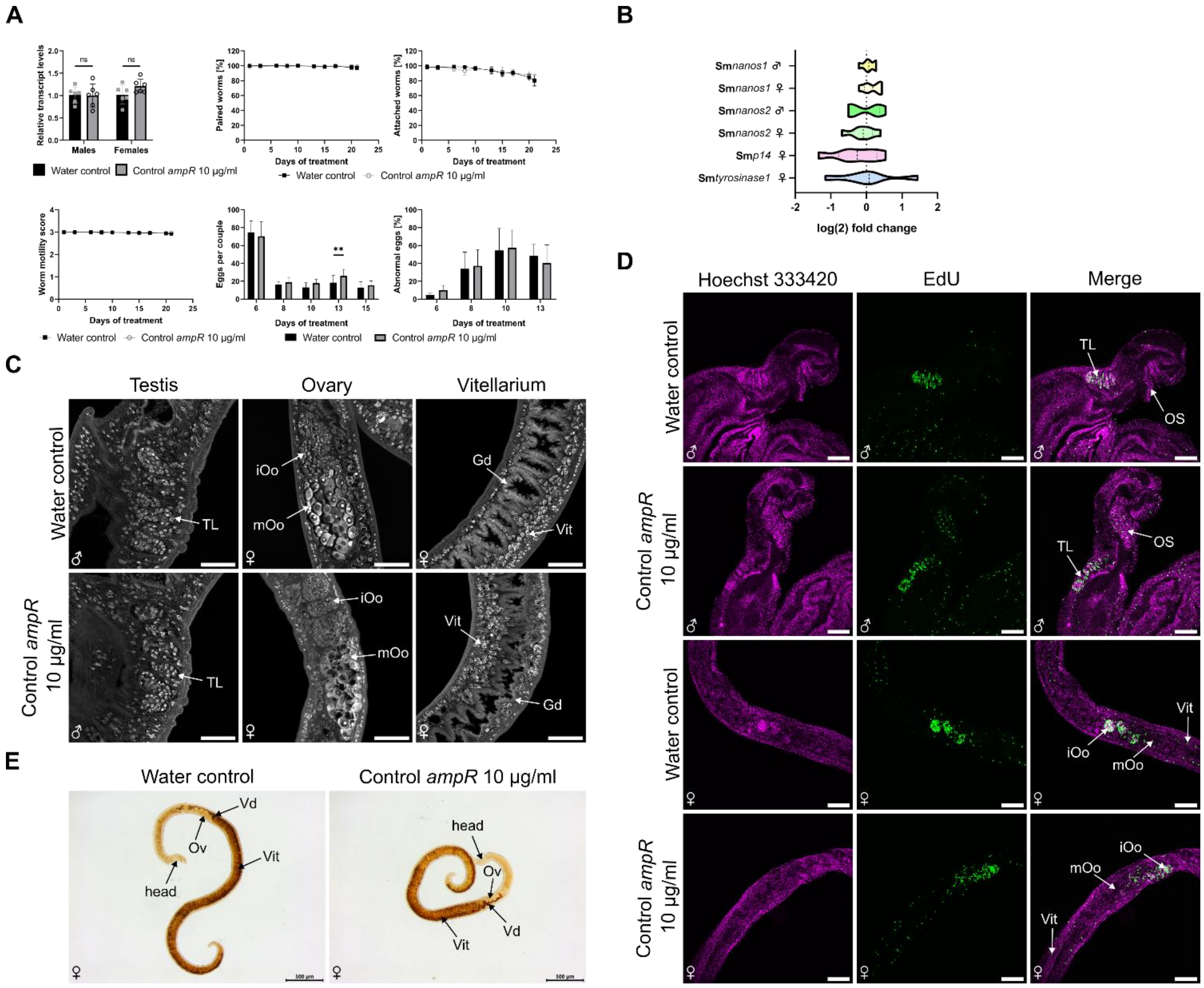

Supplemental Figure S10: Non-schistosomal dsRNA (*ampR*) at 10 µg/ml showed no effects on worm viability, morphology, stem-cell proliferation, and the expression of selected genes. **(A)** Transcript levels of Sm*eif4a-b* at d 21, and the scoring parameters pairing stability, attachment and motility were unaffected by addition of *ampR* dsRNA. Egg production was significantly higher 13 d after treatment, while the ratio of abnormal to normal eggs did not differ compared to the water control. ** $p < 0.01$, determined by *t*-test. Except for egg numbers (n = 3), remaining data are n = 6. **(B)** Transcript levels of investigated genes were normalized using the housekeeping gene Sm*letm1* (Haeberlein et al., 2019), and were similar to the water control. **(C)** CLSM analysis showed normal morphologies of the reproductive organs of males and females. **(D)** EdU assays revealed comparable stem-cell proliferation in GSCs and SSCs between the *ampR* and water control. **(E)** Lipid staining of female *S. mansoni* was unaffected by incubation with *ampR* dsRNA. Scale bars: C, 50 µm; D, 100 µm. Gd = Gastrodermis, iOo = oogonia, mOo = mature oocyte, Ov = Ovary, SV = sperm vesicle, TL = testicular lobes, Vd = vitelloduct, Vit = vitellarium. B, means ± SD of 6 independent experiments (n = 6) are shown. C-E, representative images of 3 independent experiments (n = 3) with 3 (D, E) or 6 (C) worms per experiment, analyzed after 21 d of dsRNA treatment, are depicted.

Supplemental Table S1: Sequences used for the construction of the phylogenetic tree

Additional Document (Excel file)

Supplemental Table S2: Primers used for RT-qPCR

Additional Document (Excel file)

Supplemental Table S3: Sequences of *in vitro* transcription constructs used for dsRNA or riboprobe synthesis

Additional Document (Excel file)

Supplemental Table S4: Multiple sequence alignment of SmeIF4A and human eIF4A isoforms. Sequences were aligned using Clustal Ω.

	**SmeIF4A-a**	**SmeIF4A-b**	**SmeIF4AIII**	**HseIF4AI**	**HseIF4AII**	**HseIF4AIII**
**SmeIF4A-a**	100.00%	58.61%	53.32%	57.40%	58.42%	52.55%
**SmeIF4A-b**	58.61%	100.00%	60.93%	69.67%	69.67%	61.95%
**SmeIF4AIII**	53.32%	60.93%	100.00%	66.33%	66.08%	82.29%
**HseIF4AI**	57.40%	69.67%	66.33%	100.00%	89.66%	66.91%
**HseIF4AII**	58.42%	69.67%	66.08%	89.66%	100.00%	67.49%
**HseIF4AIII**	52.55%	61.95%	82.29%	66.91%	67.49%	100.00%

Supplemental Table S5: Sm*eif4a-a* is the dominantly transcribed eIF4A isoform, especially with respect to the ovary. Based on a previous bulk RNA-seq study of adult *S. mansoni* and their gonads (Lu et al., 2016), which covered Sm*eif4a-a* and Sm*eif4a-b*, we extracted the transcript values per million units of both genes for pairing experienced (bM, bF) and pairing-unexperienced (sM, sF) males and females. Furthermore, transcript levels of Sm*eif4a-a* and Sm*eif4a-b* in isolated gonads of bM and sM (bT, sT) or bF and sF (bO, sO) are shown. The ratio of Sm*eif4a-a* transcript levels to Sm*eif4a-b* in each studied samples is shown in the last row.

Gene	bM	sM	bT	sT	bF	sF	bO	sO
Sm*eif4a-a*	407.5	380.4	917.7	965.6	505.0	426.1	1288.4	1244.3
Sm*eif4a-b*	10.5	11.0	24.4	25.7	6.4	5.9	3.90	12.5
Sm*eif4a-a* / Sm*eif4a-b*	**38.8**	**34.6**	**37.6**	**37.6**	**78.9**	**72.2**	**330.4**	**99.5**

References

Cogswell, A. A., Collins, J. J., Newmark, P. A., and Williams, D. L. (2011). Whole mount *in situ* hybridization methodology for *Schistosoma mansoni*. *Mol Biochem Parasitol* 178, 46–50. doi: 10.1016/j.molbiopara.2011.03.001

Haeberlein, S., Angrisano, A., Quack, T., Lu, Z., Kellershohn, J., Blohm, A., et al. (2019). Identification of a new panel of reference genes to study pairing-dependent gene expression in *Schistosoma mansoni*. *Int J Parasitol* 49, 615–624. doi: 10.1016/J.IJPARA.2019.01.006

Li, X., Weth, O., Haimann, M., Möscheid, M. F., Huber, T. S., and Grevelding, C. G. (2024). Rhodopsin orphan GPCR20 interacts with neuropeptides and directs growth, sexual differentiation, and egg production in female *Schistosoma mansoni*. *Microbiol Spectr* 12. doi: 10.1128/spectrum.02193-23

Lu, Z., Sessler, F., Holroyd, N., Hahnel, S., Quack, T., Berriman, M., et al. (2016). Schistosome sex matters: A deep view into gonad-specific and pairing-dependent transcriptomes reveals a complex gender interplay. *Sci Rep* 6, 1–14. doi: 10.1038/srep31150

Lück, S., Kreszies, T., Strickert, M., Schweizer, P., Kuhlmann, M., and Douchkov, D. (2019). siRNA-Finder (si-Fi) Software for RNAi-target design and off-target prediction. *Front Plant Sci* 10. doi: 10.3389/FPLS.2019.01023/BIBTEX

Moescheid, M. F., Lu, Z., Soria, C. D., Quack, T., Puckelwaldt, O., Holroyd, N., et al. (2025). The retinoic acid family-like nuclear receptor SmRAR identified by single-cell transcriptomics of ovarian cells controls oocyte differentiation in *Schistosoma mansoni*. *Nucleic Acids Res* 53. doi: 10.1093/nar/gkae1228

Ronquist, F., Teslenko, M., Van Der Mark, P., Ayres, D. L., Darling, A., Höhna, S., et al. (2012). Mrbayes 3.2: Efficient bayesian phylogenetic inference and model choice across a large model space. *Syst Biol* 61, 539–542. doi: 10.1093/SYSBIO/SYS029

Skinner, D. E., Rinaldi, G., Suttiprapa, S., Mann, V. H., Smircich, P., Cogswell, A. A., et al. (2012). Vasa-like DEAD-box RNA helicases of *Schistosoma mansoni*. *PLoS Negl Trop Dis* 6. doi: 10.1371/journal.pntd.0001686

Wendt, G., Zhao, L., Chen, R., Liu, C., O'Donoghue, A. J., Caffrey, C. R., et al. (2020). A single-cell RNAseq atlas of *Schistosoma mansoni* identifies a key regulator of blood feeding. *Science* 369, 1649. doi: 10.1126/SCIENCE.ABB7709
